# The kinase ATR controls meiotic crossover distribution at the genome scale in Arabidopsis

**DOI:** 10.1093/plcell/koae292

**Published:** 2024-10-29

**Authors:** Longfei Zhu, Julia Dluzewska, Nadia Fernández-Jiménez, Rajeev Ranjan, Alexandre Pelé, Wojciech Dziegielewski, Maja Szymanska-Lejman, Karolina Hus, Julia Górna, Mónica Pradillo, Piotr A Ziolkowski

**Affiliations:** Laboratory of Genome Biology, Institute of Molecular Biology and Biotechnology, Adam Mickiewicz University in Poznan, 61-614 Poznan, Poland; Laboratory of Genome Biology, Institute of Molecular Biology and Biotechnology, Adam Mickiewicz University in Poznan, 61-614 Poznan, Poland; Departamento de Genética, Fisiología y Microbiología, Facultad de Ciencias Biológicas, Universidad Complutense de Madrid, 28040 Madrid, Spain; Laboratory of Genome Biology, Institute of Molecular Biology and Biotechnology, Adam Mickiewicz University in Poznan, 61-614 Poznan, Poland; Laboratory of Genome Biology, Institute of Molecular Biology and Biotechnology, Adam Mickiewicz University in Poznan, 61-614 Poznan, Poland; Laboratory of Genome Biology, Institute of Molecular Biology and Biotechnology, Adam Mickiewicz University in Poznan, 61-614 Poznan, Poland; Laboratory of Genome Biology, Institute of Molecular Biology and Biotechnology, Adam Mickiewicz University in Poznan, 61-614 Poznan, Poland; Laboratory of Genome Biology, Institute of Molecular Biology and Biotechnology, Adam Mickiewicz University in Poznan, 61-614 Poznan, Poland; Laboratory of Genome Biology, Institute of Molecular Biology and Biotechnology, Adam Mickiewicz University in Poznan, 61-614 Poznan, Poland; Departamento de Genética, Fisiología y Microbiología, Facultad de Ciencias Biológicas, Universidad Complutense de Madrid, 28040 Madrid, Spain; Laboratory of Genome Biology, Institute of Molecular Biology and Biotechnology, Adam Mickiewicz University in Poznan, 61-614 Poznan, Poland

## Abstract

Meiotic crossover, i.e. the reciprocal exchange of chromosome fragments during meiosis, is a key driver of genetic diversity. Crossover is initiated by the formation of programmed DNA double-strand breaks (DSBs). While the role of ATAXIA-TELANGIECTASIA AND RAD3-RELATED (ATR) kinase in DNA damage signaling is well-known, its impact on crossover formation remains understudied. Here, using measurements of recombination at chromosomal intervals and genome-wide crossover mapping, we showed that ATR inactivation in Arabidopsis (*Arabidopsis thaliana*) leads to dramatic crossover redistribution, with an increase in crossover frequency in chromosome arms and a decrease in pericentromeres. These global changes in crossover placement were not caused by alterations in DSB numbers, which we demonstrated by analyzing phosphorylated H2A.X foci in zygonema. Using the *seed-typing* technique, we found that hotspot usage remains mainly unchanged in *atr* mutants compared with wild-type individuals. Moreover, *atr* showed no change in the number of crossovers caused by two independent pathways, which implies no effect on crossover pathway choice. Analyses of genetic interaction indicate that while the effects of *atr* are independent of MMS AND UV SENSITIVE81 (MUS81), ZIPPER1 (ZYP1), FANCONI ANEMIA COMPLEMENTATION GROUP M (FANCM), and D2 (FANCD2), the underlying mechanism may be similar between ATR and FANCD2. This study extends our understanding of ATR's role in meiosis, uncovering functions in regulating crossover distribution.

## Teaser

Inactivation of the serine/threonine protein kinase ATR redistributes meiotic crossovers in Arabidopsis chromosomes without altering double-strand break numbers or hotspot usage.

## Introduction

Crossover recombination, the exchange of DNA fragments between homologous chromosomes, is a crucial process during meiosis, as it ensures proper homolog segregation and enables shuffling of the genetic material from both parents (Villeneuve and Hillers 2001; Hunter 2015; Wang and Copenhaver 2018). Crossovers are initiated by programmed DNA double-strand breaks (DSBs), which are generated during the early stages of meiosis I by the conserved SPORULATION11 (SPO11) transesterase and its accessory proteins (Keeney et al. 1997; Vrielynck et al. 2016). Following end resection, extensive 3′ single-stranded DNA (ssDNA) overhangs are created to promote strand invasion of the homologous chromosome (Neale et al. 2005). Before this process can occur, ssDNA is coated with heterotrimeric REPLICATION PROTEIN A (RPA) followed by the recruitment of two conserved strand-exchange recombinases, RADIATION SENSITIVE51 (RAD51) and meiosis-specific DISRUPTED MEIOTIC CDNA1 (DMC1) (Shinohara et al. 1992, 1997; Bishop 1994). Both RAD51 and DMC1 are required for invasion; however, their functions differ, which is manifested by the different behaviors of their mutants in Arabidopsis (*Arabidopsis thaliana*): The *rad51-*null mutant shows extensive chromosome fragmentation due to its inability to repair meiotic DSBs. In contrast, the *dmc1* mutant fails to undergo synapsis or form crossovers but does not exhibit chromosome fragmentation because meiotic DSBs are repaired using sister chromatid (Couteau et al. 1999; Li et al. 2004; Pradillo et al. 2012, 2014; Emmenecker et al. 2023). Therefore, loading these two proteins onto the ssDNA nucleofilament is instrumental for strand invasion. In most eukaryotes, a majority of the resulting displacement loop (D-loop) intermediates are disassembled by DNA helicases and successively repaired as noncrossovers (NCOs) (Hunter 2015; Pyatnitskaya et al. 2019). A subset of D-loops undergoes second-end capture, forming different types of joint molecules, including double Holliday junctions (dHJs). The dHJ intermediates are often subjected to helicase-mediated dissolution and repaired as NCOs. In plants, NCO pathways are stimulated mainly via the activity of FANCONI ANEMIA COMPLEMENTATION GROUP M (FANCM) and RECQ4 helicases (Crismani et al. 2012; Girard et al. 2015; Séguéla-Arnaud et al. 2015; Fernandes et al. 2017).

In many eukaryotes, including plants, some dHJs are protected from helicase-mediated dissolution and enter the major crossover pathway called Class I or ZMM pathway. Class I crossovers are interfering crossovers, which means that the chance of two crossovers forming in close proximity to each other on a chromosome is lower than that resulting from a random distribution. This recombination pathway is meiosis specific and depends on proteins collectively called ZMM (after budding yeast proteins involved in this pathway). Since approximately 85% of crossovers are caused by the ZMM pathway in Arabidopsis, inactivation of any of the ZMM-encoding genes leads to crossover scarcity, resulting in random chromosome segregation and a dramatic decrease in fertility (Mercier et al. 2005). In addition to Class I crossovers, most eukaryotes have additional recombination pathways that are noninterfering and do not depend on ZMM proteins (Mercier et al. 2005). These pathways are based on the activity of structure-specific endonucleases, mainly MMS AND UV SENSITIVE81 (MUS81), which, when disabled in the Arabidopsis *zmm* mutant background, causes a further decrease in the number of crossovers (Berchowitz et al. 2007; Higgins et al. 2008). Independent of MUS81, FANCONI ANEMIA GROUP D2 (FANCD2) promotes noninterfering crossovers and control crossover distribution in plants (Kurzbauer et al. 2018). Of the approximately 150 to 200 DSBs formed during meiosis, only ∼8 will be repaired as Class I crossovers, and ∼1 will be repaired as Class II crossovers in wild type *A. thaliana* (Higgins et al. 2004; Mercier et al. 2005; Ferdous et al. 2012; Kurzbauer et al. 2012).

Because the formation of programmed DSBs and ssDNA threatens genome stability, these processes activate the DNA damage response (DDR) signaling network, which controls and executes key steps of DSB repair (Zhou and Elledge 2000; Zou and Elledge 2003). DDR initiation occurs through a protein phosphorylation cascade, which is primarily controlled by the ATAXIA-TELANGIECTASIA MUTATED (ATM) and ATAXIA-TELANGIECTASIA AND RAD3-RELATED (ATR) kinases (Pereira et al. 2020). While ATM is activated mainly by unprocessed DSBs, ATR activation is induced by the generation of ssDNA resulting from DSB resection and its subsequent stabilization by RPA (Maréchal and Zou 2013).

ATR plays diverse roles by phosphorylating a large group of proteins with various functions; however, these roles of ATR are best studied in the context of DNA repair in somatic cells (Pereira et al. 2020). In meiosis, ATR participates in the control of DSBs to a lesser extent than ATM does (Cooper et al. 2014; Garcia et al. 2015; Li and Yanowitz 2019). Unlike ATM-deficient mice, which have an increased number of meiotic DSBs, ATR-deficient mice exhibit no visible changes in DSB frequency (Pacheco et al. 2018; Widger et al. 2018). However, in lines in which both kinases are deficient, the increase in DSBs exceeds that in lines with ATM deficiency alone (Widger et al. 2018). This is similar to what has been observed in *A. thaliana*, where the *atm* mutant shows strong chromosome fragmentation during meiosis and a decrease in fertility; the *atr* mutant does not show any visible disturbances in meiosis or a decrease in fertility; and the *atr atm* double mutant is completely sterile (Garcia et al. 2003; Culligan et al. 2004; Culligan and Britt 2008). Interestingly, *atr atm* sterility is related to the inability of this mutant to initiate synapsis, indicating the early function of both kinases in the formation of the ssDNA nucleofilaments necessary for chromosome pairing in Arabidopsis (Grelon et al. 2001; Culligan and Britt 2008). Consistent with this observation, *atr* and *atm* mutants exhibit altered loading of DMC1 and RAD51 recombinases on ssDNA at the initial stages of DSB repair (Kurzbauer et al. 2012, 2021). Moreover, a drastic, more than 2-fold increase in the number of DMC1 foci and a moderate decrease in the number of RAD51 foci were observed in *atr* mutants (Kurzbauer et al. 2012). However, it remains unclear whether the total number of DSBs is altered in Arabidopsis *atr* mutants. It is also not known whether the loss of ATR has any impact on crossover formation, frequency, or chromosomal position.

Here, we show that Arabidopsis *ATR* knockout leads to genome-wide crossover redistribution, as indicated by an increase in the crossover rate in chromosome arms and a reciprocal decrease in pericentromeres. However, this difference in crossover pattern is likely not caused by an increase in the number of DSBs since the number of γH2A.X foci are not changed in *atr*. In the *atr* mutant, the number of MLH1 foci indicating Class I crossover sites did not change, and crossover interference was not affected. Moreover, the *atr* mutation does not lead to an increase in Class II crossovers, as we demonstrated by analyzing the number of bivalents and fertility in the *atr zip4* mutant. While investigating the *atr* crossover topology at the fine scale, we observed a similar hotspot usage as in wild type. Furthermore, we found that *atr* and *fancd2* mutations cause similar changes in crossover distribution, possibly through a shared mechanism but triggered via different pathways. Finally, we showed that the changes in crossover frequency observed in *atr* mutants did not depend on the pattern of heterozygosity across the chromosome. Our results suggest that ATR controls crossover formation at the early stages of meiosis and are therefore largely independent of the activity of individual crossover pathways.

## Results

### Loss of ATR function leads to local changes in crossover frequency

**Figure 1.**
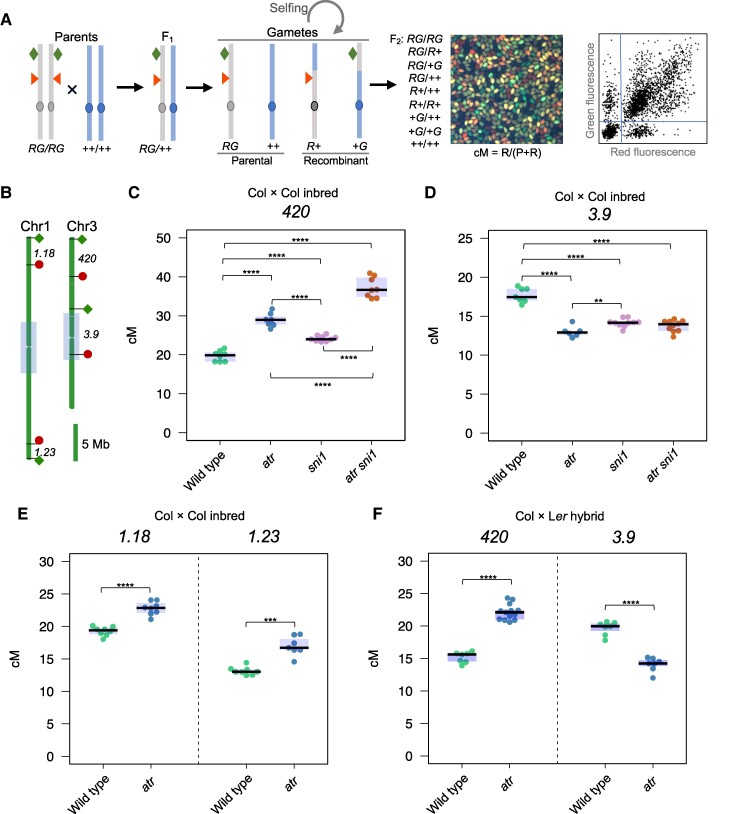
Disruption of *ATR* results in changes in the local crossover frequency. **A)** Diagram showing the measurement of crossover rates using a seed-based FTL system. FTL carries red (R) and green (G) fluorescent reporters encoded on one chromosome (red triangle and green square) that define the genetic interval. After crossing FTL (RG/RG) with a nonfluorescent *A. thaliana* line (++/++), we obtained F_1_ plants that were hemizygous for the reporters (RG/++) and produced gametes containing parental or recombinant reporter combinations. F_1_ plants were left to self-pollinate, and F_2_ seeds of different genotypes were produced and visualized under a fluorescent stereomicroscope. Pictures of F_2_ seeds in red/green fluorescent channels were taken, and analysis via CellProfiler allowed us to precisely measure the crossover frequency within the interval. cM, centimorgan; R, recombinant; P, parental. **B)** The ideograms of chromosomes 1 and 3 with the location of the *1.18*, *1.23*, *420*, *and 3.9* intervals in relation to the pericentromeres defined as regions with higher than average DNA methylation, which surround the centromeres (gray boxes). Green squares and red circles indicate the positions of eGFP and dsRed reporters used to measure crossover frequency. **C)** Crossover frequency (cM) within the *420* interval in the Col × Col inbred line for the wild type, *atr*, *sni1,* and *atr sni1*. The center line of a boxplot indicates the median; the upper and lower bounds indicate the 75th and 25th percentiles, respectively. **D)** Same as in B but for the *3.9* interval. **E)***1.18* and *1.23* crossover frequencies (cM) in the Col × Col inbred line for the wild type and *atr*. **F)***420* and *3.9* crossover frequencies (cM) in the Col × L*er* hybrid for the wild type and *atr*. Significance in **C–D** was calculated using one-way ANOVA with Tukey HSD; significance in **E**-**F** was assessed by Welch *t*-tests; *P < 0.05, **P < 0.01, ***P < 0.001, ****P < 0.0001. Not significant *P*-values were not shown. Each dot represents a measurement from one individual.

We previously identified *SUPPRESSOR OF NPR1-1 INDUCIBLE1* (*SNI1*) as a natural modifier of meiotic crossover in Arabidopsis, the mutation of which results in an increased Class II crossover frequency in subtelomeric chromosomal regions (Zhu et al. 2021). Inactivation of the *ATR* gene could partially suppress the retarded growth of *sni1* (Yan et al. 2013). Therefore, we tested whether the *atr* mutant can also suppress the meiotic crossover phenotype of *sni1*. For this purpose, we chose to analyze crossovers via an assay based on the segregation of fluorescent reporter genes in the background of fluorescently tagged lines (FTLs) (Fig. 1A) (Melamed-Bessudo et al. 2005; Ziolkowski et al. 2015; Kbiri et al. 2022). We measured the crossover frequency of the wild type, *atr*, *sni1*, and *atr sni1* in *420* and *3.9* intervals, which cover the subtelomeric and pericentromeric regions of chromosome 3 (Fig. 1B). We previously observed an increase in crossover frequency in *sni1 atr* double mutants within the *420* interval compared with *sni1* (37.2 cM vs. 28.9 cM in *sni1 atr* and *sni1*, respectively; one-way ANOVA followed by Tukey HSD *P* = 2.6 × 10^−9^; Fig. 1C, Supplementary Table S1) (Zhu et al. 2021). However, *sni1 atr* and *sni1* exhibited similar *3.9* crossover frequencies (14.1 cM vs. 13.7 cM in *sni1 atr* and *sni1*, respectively; *P* = 0.196), which was significantly lower than that of the wild type (17.7 cM; *P* < 9.2 × 10^−12^; Fig. 1D, Supplementary Table S2). This result suggested that inactivation of *ATR* does not suppress the increase in meiotic crossover in *sni1*. Surprisingly, we found that, compared with wild-type plants, the *atr* mutant exhibited a significantly greater crossover frequency in the *420* interval (24.2 cM; *P* = 5.7 × 10^−5^) and a significantly greater reduction in *3.9* (14.1 cM; *P* = 7.8 × 10^−11^) (19.7 cM and 17.7 cM for *420* and *3.9*, respectively) (Fig. 1, C and D, Supplementary Tables S1 and S2).

To exclude the possibility that crossover frequency changes are specific to Chromosome 3, we backcrossed the *atr* mutant to two FTLs located in subtelomeric regions of Chromosome 1, *1.18* and *1.23* (Fig. 1B). As in the *420* interval, we observed a significantly increased crossover frequency in both intervals in these plants compared to that in the wild type (from 19.26 to 22.78 and from 13.16 to 17.00 cM for *1.18* and *1.23*, respectively; Fig. 1E, Supplementary Table S3). Taken together, these data suggest local changes in the crossover distribution in *atr*.

### *Atr* shows global remodeling of the meiotic crossover landscape

Since DNA polymorphisms between homologs affect crossover distribution, the inactivation of some genes may influence recombination differently in inbreds (completely homozygous genome) and in hybrids (completely heterozygous genome). For example, the *Arabidopsis fancm* mutation causes a substantial increase in crossover frequency in inbred plants, while in hybrids, crossover frequency does not change (Crismani et al. 2012; Girard et al. 2015; Ziolkowski et al. 2015). To investigate the effects of *ATR* inactivation in *A. thaliana* hybrids, we first generated an *atr* knockout mutant in the *A. thaliana* L*er*-0 accession (hereafter L*er*) using CRISPR-Cas9 (Supplementary Fig. S1). Then, we crossed this allele with the *atr* mutant allele in the reference accession Col-0 (hereafter referred to as Col), and in the obtained Col/L*er atr* hybrids, we measured the crossover frequency using FTL intervals. The *420* crossover frequency in the Col/L*er* hybrids increased from 15.82 cM in the wild type to 22.13 cM in the *atr* mutant (*P* = 4.0 × 10^−11^; Fig. 1F, Supplementary Table S4). However, the *3.9* crossover frequency in hybrids decreased from 19.66 cM in the wild type to 14.08 cM in the *atr* mutant (*P* = 4.5 × 10^−7^; Fig. 1F, Supplementary Table S4). These changes are very similar to those observed in Col/Col inbred plants (Fig. 1, C and D). We therefore concluded that the crossover remodeling observed in *atr* is not inhibited by interhomolog polymorphisms.

**Figure 2.**
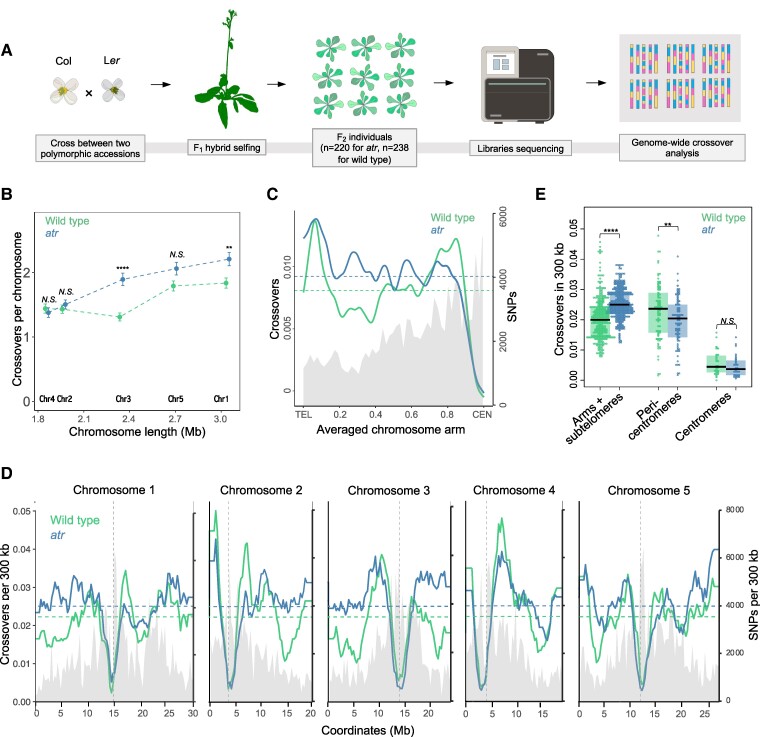
The *atr* mutant exhibits a global redistribution of meiotic crossovers. **A)** Scheme explaining the identification of genome-wide crossover events via genome sequencing. The identification of crossover sites was carried out based on genotype transitions in each F_2_ individual detected via SNPs differing from those in the Col and L*er* parents. **B)** Differences in total crossover numbers for each chromosome in *atr* vs. wild type. Mean crossover numbers are shown, while error bars define SEs. The Mann‒Whitney *U* test was used to calculate statistical significance; *P < 0.05, **P < 0.01, ***P < 0.001, ****P < 0.0001. **C)** Crossover frequency per 300 kb per F_2_ along the proportional (scaled) length of the chromosomes from telomeres (TEL) to centromeres (CEN). The mean values are shown by horizontal dashed lines. Col/L*er* SNPs per 300 kb are plotted and shaded in gray. **D)** Data are the same as those in **C** but showing the crossover frequency and SNP density plotted along five Arabidopsis chromosomes. The position of each centromere is indicated by the vertical dashed line. **E)** A cumulative analysis of crossover frequency in *atr* compared with wild type for chromosome arms, pericentromeres, and centromeres. Each dot represents a single 300 kb window from **D**. The center line of a boxplot indicates the median; the upper and lower bounds indicate the 75th and 25th percentiles, respectively. Centromeres are defined by a higher-than-average nucleosome density, while pericentromeres, which flank the centromeres, are defined by regions with elevated DNA methylation levels. *P*-values were calculated as in **B**.

To explore the extent to which the observed crossover changes were global, we mapped crossovers at the genome-wide scale. We used genome sequencing data from 220 F_2_ individuals produced from selfing *atr* Col/L*er* hybrids (Fig. 2A, Supplementary Table S5). Crossovers were identified as genotype switches along the chromosomes and were assigned to the midpoint between SNPs. In parallel, we prepared a crossover map based on 238 wild-type Col/L*er* individuals. In total, we identified 2,016 *atr* Col/L*er* crossovers, which were compared with the 1,922 crossovers for the wild-type Col/L*er*. This analysis showed that *atr* had more crossovers per individual than did the wild type (9.16 and 8.08 for *atr* and the wild type, respectively; *P* = 9.6 × 10^−6^; Mann‒Whitney *U* test; Supplementary Fig. S2). Interestingly, we observed that the increase in crossover number occurred mostly for long metacentric Chromosomes 1 and 3 (Fig. 2B; Mann‒Whitney *U* test *P* = 7.1 × 10^−3^ and 6.2 × 10^−6^ for Chromosomes 1 and 3, respectively). For all five chromosomes, there was a clear redistribution of crossovers from pericentromeric regions to interstitial and subtelomeric regions (Fig. 2, C and D). A cumulative analysis of chromosome arms revealed a significantly higher crossover frequency in atr compared with wild type, while an opposite trend was observed in pericentromeric regions (Fig. 2E; Mann‒Whitney *U* test *P* < 2.2 × 10^−16^ and *P* = 0.01393, respectively). Centromeres are defined by a higher-than-average nucleosome density, while pericentromeres, which flank the centromeres, are defined by regions with elevated DNA methylation levels (Choi et al. 2018). This result confirms our observation based on FTL intervals that there is extensive remodeling of the crossover landscape in *atr*, which is associated with a modest increase in crossover frequency.

### The activity of recombination hotspots remains similar in *atr* compared with wild type

Genome-wide crossover analysis does not provide the resolution to examine the extent to which the observed changes translate to the level of recombination hotspots. To investigate this phenomenon, we decided to use the *seed-typing* technique recently developed by our team (Szymanska-Lejman et al. 2023). Briefly, *seed-typing* is based on the use of fluorescently labeled extremely short interval lines (ESILs; < 50 kb). A cross between the selected ESIL in the Col and L*er* lines led to the creation of a Col/L*er* hybrid, which produced seeds segregating for eGFP and dsRed reporters when left to self-pollinate. This allows us to precisely measure the crossover frequency within the interval in the same way as for regular FTL lines. The recombinants were then manually selected, and the interval at which the crossover occurred was amplified by high-fidelity long-range PCR using overlapping amplicons. The PCR products were used to construct Illumina-compatible libraries, separately for each recombinant, and then sequenced to a depth of ∼1500×. For each recombinant, we determined the crossover site based on SNPs differentiating Col from L*er*. Superimposing the crossover placement from multiple recombinants allows us to map the distribution of crossovers at the fine scale.

**Figure 3.**
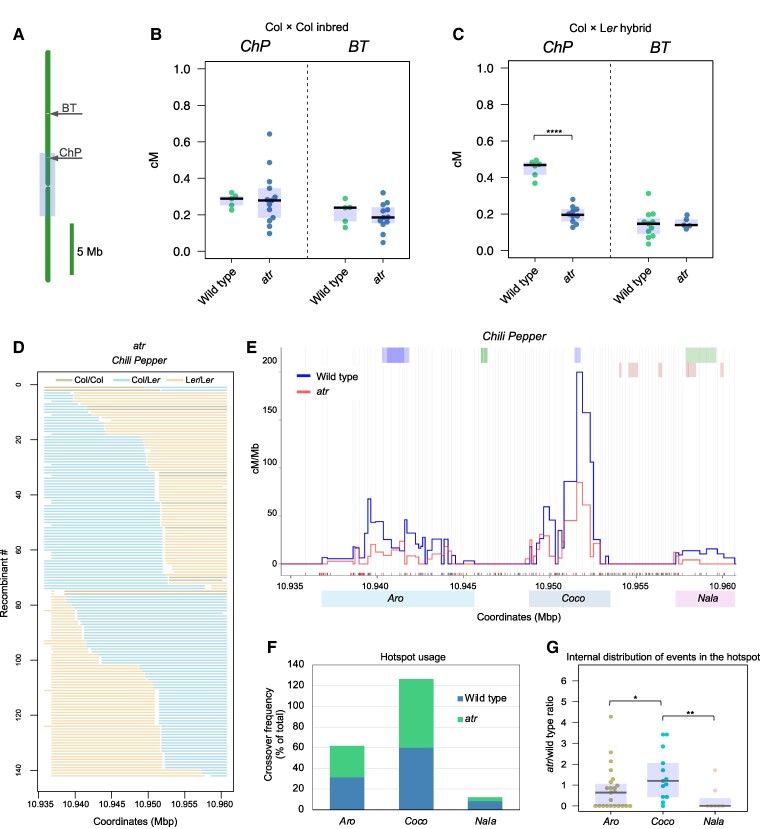
The activity of *ChP* crossover hotspots remains similar in *atr* compared with wild type. **A)** The ideogram of Chromosome 3 with the location of the *ChP* and *Bow Tie* (*BT*) intervals in relation to the pericentromere defined as a region with higher than average DNA methylation, which surrounds the centromere (gray box). **B)***ChP* and *BT* crossover frequency (cM) in Col × Col inbred plants for the wild type and *atr*. The center line of a boxplot indicates the median; the upper and lower bounds indicate the 75th and 25th percentiles, respectively. Each dot represents a measurement from one individual. **C)** Same as in **B** but in the Col × L*er* hybrid. Significance in **B**-**C** was assessed by Welch *t*-tests; ****P < 0.0001, not significant *P*-values were not shown. **D)** Genotypes of 142 Col-*ChP* × L*er atr* recombinants along the *ChP* interval. Each horizontal line corresponds to one recombinant with a color-encoded genotype. **E)** Crossover landscape within *ChP* for the Col-*ChP* × L*er* wild type (blue) and Col-*ChP* × L*er atr* (red). Crossovers are normalized to the *ChP* crossover frequency shown in C. SNPs spaced at least 100 bp apart, shown as vertical gray lines, were used to determine the crossover topology. The black and red *x*-axes correspond to all the Col/L*er* SNPs and InDels, respectively. Genes are shown as light-green (forward) and dark-green (reverse) rectangles, and transposons are shown as blue rectangles. The wild-type data were obtained from Szymanska-Lejman et al. (2023). **F)***ChP* hotspot usage for *atr* (*n* = 142) and wild type (*n* = 243). The chi-square test showed no deviation in the hotspot distribution in *atr* compared with wild type. **G)** Relative hotspot activity represented as an *atr*/wild-type crossover rate ratio. Each dot shows the ratio calculated for a single pair of SNP section of a hotspot. Boxplots defined as in **B**. Kruskal–Wallis test followed by post hoc Dunn's test was used to assess statistical significance of the difference between the groups; *P < 0.05, **P < 0.01.

We backcrossed the *atr* mutant into two previously described ESILs, *Chili Pepper* (*ChP*), which is located in the pericentromeric region of Chromosome 3, and *Bow Tie* (*BT*), which is located in the middle of the long arm of this chromosome (Fig. 3A). The numbers of crossovers in the inbred context for *atr* did not significantly differ from those for the wild type at either interval (Fig. 3B, Supplementary Table S6). In the Col/L*er* hybrids, the crossover rate remained unchanged in *BT* but decreased significantly in *ChP* compared with that in the wild type (Fig. 3C, Supplementary Table S7). Therefore, we chose to investigate the crossover distribution in the *ChP* interval using *seed-typing*. In total, we managed to identify crossovers in 142 recombinants (Fig. 3D, Supplementary Table S5).

The *ChP* interval contains three clearly separated hotspots: *Aro*, *Coco*, and *Nala*. Although all three hotspots remained active in *atr*, we observed decreases in their activity (Fig. 3E). We then compared the percentage contribution of individual hotspots to the crossover activity of the entire interval. We observed that the strongest hotspot, *Coco*, had a 6.0% increase in its contribution to the total *ChP* crossover rate, while the contributions of the *Aro* and *Nala* hotspots decreased by 1.3% and 4.8%, respectively, compared with the wild type (Fig. 3F). Although the hotspot usage was not different from that observed in wild type (chi-square test *P* = 0.181), we found differences in the distribution of events within individual hotspots. In this analysis, each hotspot was divided into sections defined by a pair of neighboring SNPs and for each section we counted the number of crossovers normalized to the number of sequenced samples for *atr* and wild type. We then plotted the *atr*-to-wild type crossover ratio for each SNP-SNP section in all three hotspots; for *Aro*, *Coco*, and *Nala,* the mean ratios were 0.79, 1.46, and 0.35, respectively. We concluded that the *ChP* hotspot usage is similar in the *atr* mutant compared with wild type, however the internal distribution of crossover events within hotspots may differ.

### Meiosis progression is not affected in *atr*

Alterations in the pattern of crossover placement along the chromosome may be due to impaired homolog pairing and bivalent formation. For instance, in heterozygous *asy1/+* mutants, in which the level of the chromosome axis ASYNAPTIC1 (ASY1) protein is decreased, there is a dramatic redistribution of crossovers from pericentromeres to subtelomeric regions, while the total number of crossovers was not changed (Lambing et al. 2020). Therefore, we characterized the effect of the *atr* mutation on meiosis progression by studying the chromosome spreads of pollen mother cells followed by DAPI staining. We did not observe any abnormalities during meiotic progression after analyzing 134 and 177 cells in the first and second meiotic division, respectively, from three different plants: All chromosomes formed perfectly synapsed bivalents in pachynema, five bivalents were invariably detected at metaphase I, and no chromosome fragmentation was observed (Supplementary Fig. S3), which is consistent with previous reports (Culligan and Britt 2008; Kurzbauer et al. 2012). This finding suggested that the loss of *ATR* does not lead to detectable disturbances at any stage of meiosis in Arabidopsis.

### The observed changes in the *atr* crossover pattern are not associated with changes in DSB number

**Figure 4.**
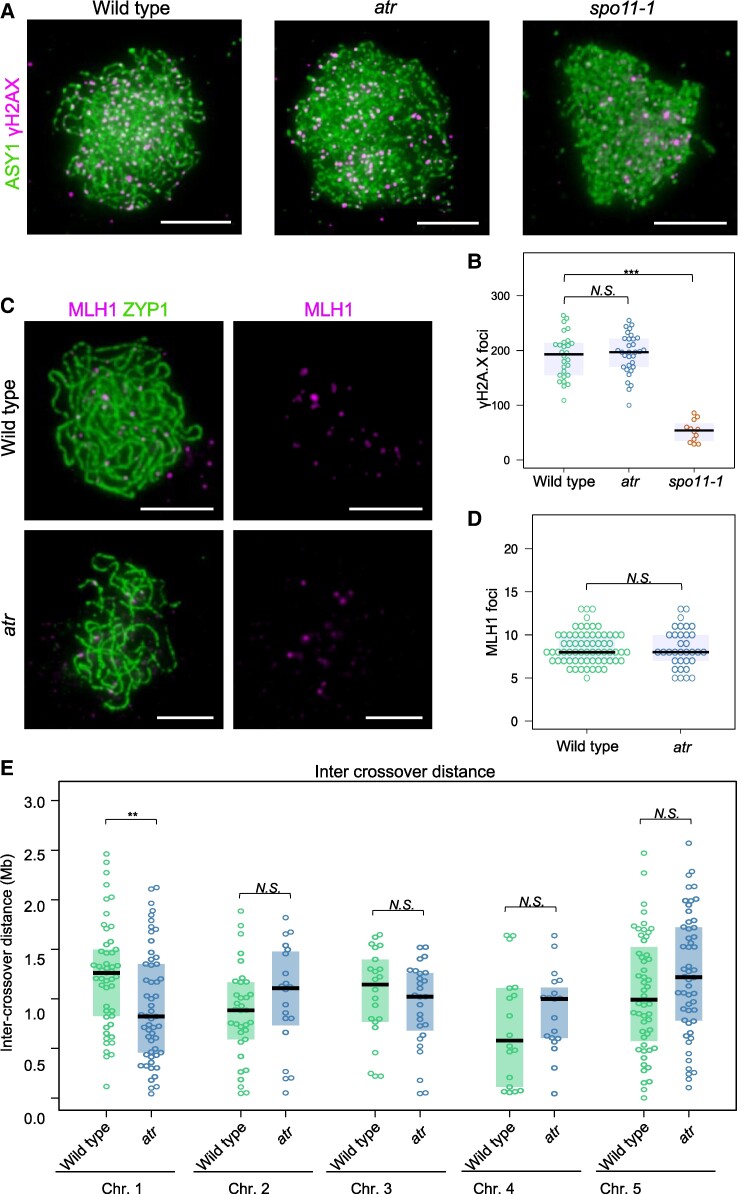
The number of γH2A.X and MLH1 foci was not changed in *atr*. **A)** Representative images of zygotene chromosomes immunostained for ASY1 (green) and γH2A.X (pink) in the wild type, *atr* and *spo11-1* (negative control). Scale bars, 5 µm. **B)** Quantification of γH2A.X focus counts in the wild type vs. *atr*. Each dot represents a measurement from one cell. The center line of a boxplot indicates the median; the upper and lower bounds indicate the 75th and 25th percentiles, respectively. Significance was assessed using one-way ANOVA with Tukey HSD; ***P < 0.001. **C)** Representative images of pachytene-stage male meiocytes from the wild type or *atr* plants stained for MLH1 (pink) and ZYP1 (green). Scale bars, 5 μm. **D)** Same as in **B** but for MLH1 foci. The Mann‒Whitney *U* test was used to calculate statistical significance. Boxplots defined as in **B**. *N.S.*, not significant. **E)***Cis*-intercrossover distances calculated from parental–heterozygous–parental genotype transitions separately for each chromosome. The center line of a boxplot indicates the median, while the upper and lower bounds indicate the 75th and 25th percentiles, respectively. Each dot represents one *cis*-intercrossover distance. Significance was assessed using the Mann–Whitney *U* test; **P < 0.01, *N.S.*, not significant.

ATR is involved in DSB signaling; therefore, one possible reason for the increased number of crossovers and their different distributions in the *atr* mutant may be an increase in the number of meiotic DSBs. For instance, Arabidopsis *spo11-1* hypomorphs with decreased DSB numbers exhibit reduced chiasma counts and altered patterns of crossover distribution (Xue et al. 2018). Since there are no methods for direct quantitative analysis of meiotic DSBs in *A. thaliana*, it is common to use the number of foci formed by proteins involved in DSB repair, such as the phosphorylated histone H2A.X (γH2A.X) or the recombinases DMC1 and RAD51, as a proxy (Bishop et al. 1992; Shinohara et al. 1992; Rogakou et al. 1998; Kurzbauer et al. 2012). Previously, Kurzbauer et al. (2012) reported a substantial, more than 2-fold increase in the number of DMC1 foci in *atr* compared with wild type, with a simultaneous >20% reduction in the number of RAD51 foci. This result indicates that DMC1/RAD51–ssDNA filament formation is affected by *atr*, but it is unclear whether this is due to a change in DSB numbers or an inhibitory effect of ATR on DMC1 loading onto the nucleofilament. We therefore decided to count the γH2A.X foci that precede the recruitment of recombinases at the DSB site. We detected 193.6 (±6.5, *n* = 32) γH2A.X foci in *atr*, which was not significantly different from the 189.9 (±7.6, *n* = 28) foci detected in the wild type (Mann–Whitney *U* test *P* = 0.609; Fig. 4, A and B). As a negative control, we used the *spo11-1* mutant, in which we detected only 53 (±5.6, *n* = 12) foci. These results suggest that ATR does not affect the number of DSBs formed during meiosis.

### Mutation in *ATR* does not affect the number of MLH1 foci or crossover interference

Since we did not observe changes in the number of DSBs that could explain the increase in the frequency and redistribution of crossovers, we investigated whether the *atr* mutation affects the number of interfering Class I crossovers. We performed MLH1 immunostaining on pachytene-stage male meiocytes. MLH1 is a component of the MutLγ endonuclease involved in DSB repair in the ZMM pathway, and it is generally accepted that the number of MLH1 foci in pachynema corresponds to the number of Class I crossovers (Chelysheva et al. 2010). On average, we observed 8.47 (±0.39, *n* = 34) MLH1 foci in *atr*, which was not significantly different from the 8.56 (±0.23, *n* = 66) foci in wild-type Col (Mann‒Whitney *U* test *P* = 0.846) (Fig. 4, C and D). This result suggested that ATR does not affect the Class I crossover number.

Class I crossovers are interfering crossovers, which means that two crossover events are located further apart on the chromosome than would be expected from a random distribution (Wang and Copenhaver 2018). Class II crossovers do not have this property; hence, their position on the chromosome is independent. If the Class I and Class II crossover ratios are disturbed *atr*, interference should be affected. Genome sequencing data can be used to estimate the level of interference by examining the distance between two crossovers that occurred during meiosis on the same chromosome. We therefore used our Col × L*er atr* F_2_ genome sequencing data to perform *cis*-double crossover (cis-DCO) analysis by filtering for parental–heterozygous–parental genotype transitions (i.e. Col-Het-Col or Ler-Het-Ler) (Drouaud et al. 2006; Sun et al. 2019) and measuring *cis*-DCO distances. We compared the *atr* intercrossover distances for each chromosome individually and observed that they were not different from those of the wild type (Fig. 4E) (Mann‒Whitney *U* test). This result suggested that crossover interference is not affected in *atr*.

### The total number of noninterfering crossovers was not altered in the *atr* mutant

**Figure 5.**
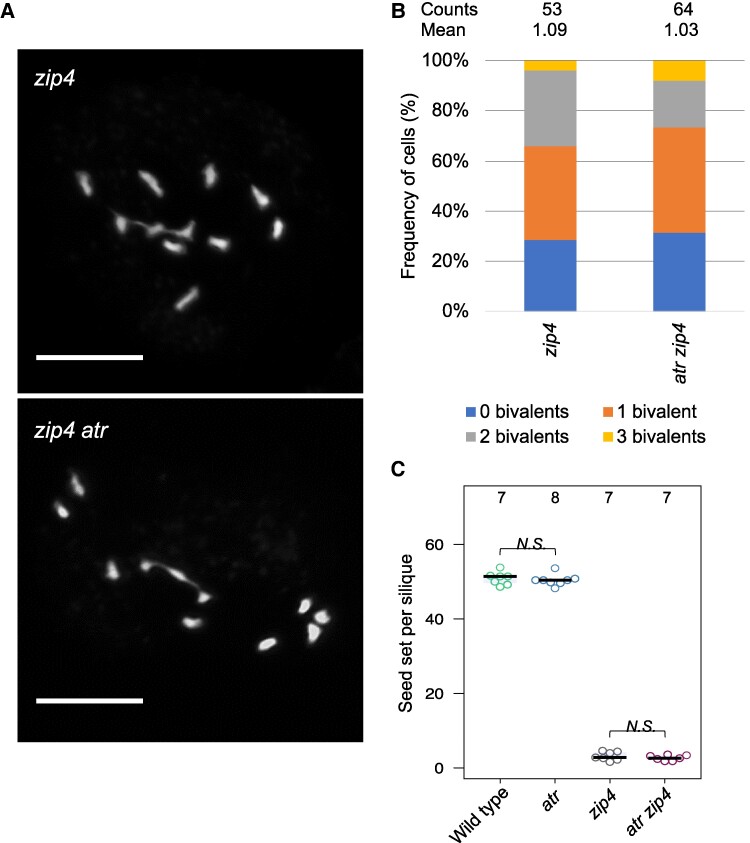
Mutation of *ATR* does not increase the chiasma number in *zip4* or restore its fertility. **A)** Representative pictures of DAPI-stained chromosomes of *zip4* and *atr zip4* at metaphase I. Scale bar, 10 μm. **B)** Quantification of univalents and bivalents in *zip4* and *atr zip4* at metaphase I. Cells were classified according to univalent/bivalent counts. The mean bivalent number per cell and the number of cells analyzed are indicated above each bar. **C)** Seed set of the wild type, *atr*, *zip4*, and *atr zip4* plants. The center line of a boxplot indicates the median; the upper and lower bounds indicate the 75th and 25th percentiles, respectively. Each dot represents a measurement from five siliques of one plant. The numbers of individuals are indicated above the boxplots. One-way ANOVA with Tukey HSD was used to estimate statistical significance; *N.S.*, not significant.

It is possible that in the case of a small increase in the number of Class II crossovers, we would not be able to observe a change in the interference level using a method based on *cis*-DCO analysis. Therefore, we decided to use additional approaches that would exclude changes to the level of Class II crossovers. Unfortunately, there are no reliable Class II crossover markers available. Instead, we tested whether the loss of ATR leads to an increase in bivalent counts in *zmm* mutants, which exhibit numerous univalents as a result of major crossover pathway (ZMM pathway) blockage (Chelysheva et al. 2007). Therefore, we crossed *atr* mutants with *zip4* knockout mutants and counted the bivalents at metaphase I. No significant differences were observed between the *zip4* and *atr zip4* mutants; on average, 1.09 bivalents were observed in *zip4* (*n* = 53), while 1.03 in *atr zip4* (*n* = 64) (Fig. 5, A and B). We did not observe an improvement in plant fertility, as measured by seed count, between these two mutants (Fig. 5C, Supplementary Table S8). Our data suggest that there are neither an increase nor reduction in Class II crossovers in the *atr* mutant.

### Crossover remodeling in *atr* occurs independently of ZMM pathway activity

We investigated whether *atr*-dependent crossover remodeling can occur in *zip4* mutants when the interfering Class I crossover pathway is abolished. As mentioned before, in the absence of the ZIP4 protein, only Class II crossovers are formed, but the number of these crossovers is too low to ensure proper chromosome segregation during meiosis and plant fertility (Chelysheva et al. 2007). Aberrant meiotic chromosome segregation prevents reliable measurement of crossover frequency in seed-based FTL systems. To restore fertility in *zip4* mutants, it is necessary to concurrently elevate the occurrence of Class II crossovers. This can be achieved by disabling FANCM helicase (Crismani et al. 2012; Knoll et al. 2012). Hence, we crossed *atr* mutants with *fancm zip4* double mutants. For crossover frequency measurements, we used two chromosomal intervals, subtelomeric *420* and pericentromeric *3.9*, which showed opposite changes in recombination frequency in *atr* compared with wild type (see Fig. 1, B and C).

**Figure 6.**
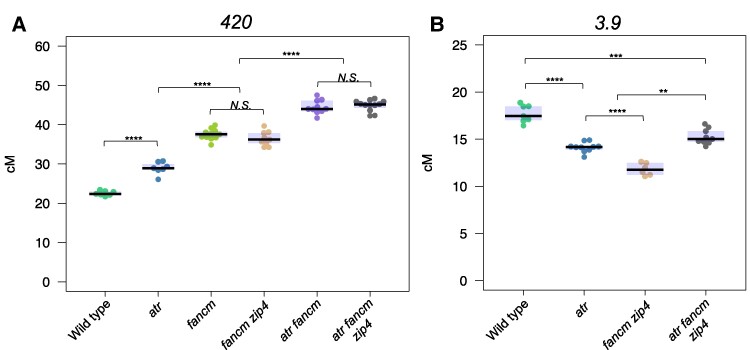
The remodeling of crossover distribution in *atr* occurs irrespective of ZMM pathway activity. **A)***420* crossover frequency (cM) in the wild type, *atr*, *fancm*, *fancm zip4*, *atr fancm*, and *atr fancm zip4* plants. The center line of a boxplot indicates the median; the upper and lower bounds indicate the 75th and 25th percentiles, respectively. Each dot represents a measurement from one individual. Significance was calculated using one-way ANOVA with Tukey HSD; **P < 0.01, ***P < 0.001, ****P < 0.0001, *N.S.*, not significant. **B)** As in **A**, but for the *3.9* interval in the wild type, *atr*, *fancm zip4*, and *atr fancm zip4* plants.

As expected, compared with those of the *atr* and wild type, both the *fancm* and *fancm zip4* mutants presented significant increases in *420* crossover frequency (mean = 37.5, 36.5, 29.0 and 22.6 cM for *fancm*, *fancm zip4*, *atr*, and wild type, respectively; Fig. 6A; statistical test indicated in the figure). However, further increases were observed when *fancm* or *fancm zip4* were combined with the *atr* mutation (mean = 44.5 and 44.9 cM for *atr fancm* and *atr fancm zip4*, respectively; Fig. 6A, Supplementary Table S9). Consistent with previous reports, *fancm zip4* showed reduced recombination rates in the pericentromeric *3.9* interval (mean = 11.8 cM vs. 17.7 cM in the wild type; Fig. 6B, Supplementary Table S10) (Dluzewska et al. 2023). Unexpectedly, *atr fancm zip4* exhibited a significantly higher crossover frequency (15.3 cM) than *fancm zip4* and *atr* (one-way ANOVA followed by Tukey HSD *P* ≤ 0.010), although it was still lower than that of the wild type (*P* = 1.2 × 10^−6^). Taken together, these observations indicate that the redistribution of crossovers in *atr* differs from that observed in the *fancm zip4* mutant and does not depend on the activity of the ZMM pathway.

### Crossover remodeling in *atr* is MUS81-, FANCD2- and ZYP1-independent

**Figure 7.**
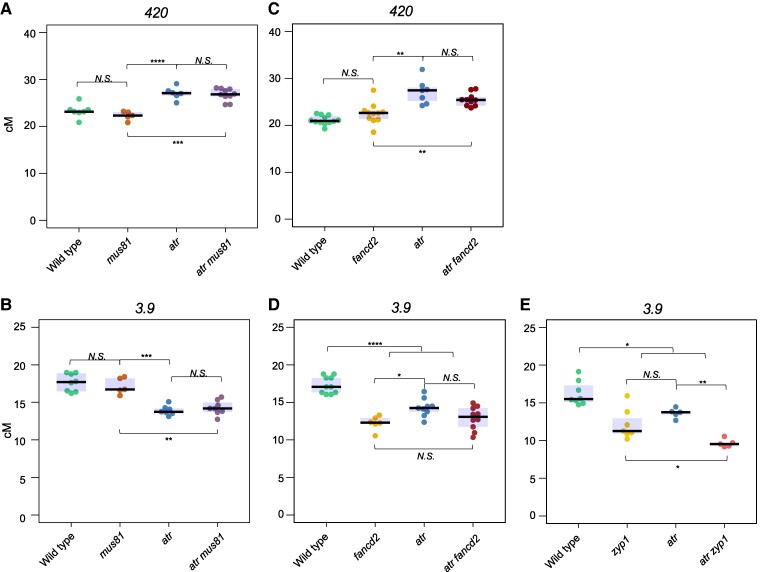
The remodeling of crossovers in *atr* is independent of MUS81, FANCD2 and ZYP1. **A)***420* crossover frequency (cM) in the wild type, *mus81*, *atr*, and *atr mus81*. The center line of a boxplot indicates the median; the upper and lower bounds indicate the 75th and 25th percentiles, respectively. Each dot represents a measurement from one individual. Significance was calculated using one-way ANOVA with Tukey HSD; *P < 0.05, **P < 0.01, ***P < 0.001, ****P < 0.0001, *N.S.*, not significant. **B)** As in **A**, but for the *3.9* interval. **C)** As in **A**, but for the wild type, *fancd2*, *atr*, and *atr fancd2*. **D)** As in **B**, but for the wild type, *fancd2*, *atr*, and *atr fancd2*. **E)** As in **A**, but for the wild type, *zyp1*, *atr*, and *atr zyp1*.

We also investigated whether the crossover remodeling observed in *atr* is controlled by the structure-specific endonuclease MUS81, which is considered a major resolvase in the noninterfering crossover pathway (Mercier et al. 2015). To this end, we backcrossed the *mus81-2* knockout mutant (Hartung et al. 2006) to the *420* and *3.9* reporter lines. It was previously shown that a *mus81* mutation in combination with a *msh4* mutation, one of the ZMM components, results in a reduced mean chiasma frequency and a moderate decrease in crossover frequency in some genetic intervals when compared with a single *msh4* mutant (Berchowitz et al. 2007; Higgins et al. 2008). We did not observe significant changes in crossover frequency in either the *420* or *3.9* interval in a single *mus81* mutant compared with the wild type (Fig. 7, A and B, Supplementary Tables S11 and S12). This is expected, as the ZMM pathway is active in these lines and is responsible for the vast majority of crossovers. However, the crossover frequency of the *atr mus81* double mutant did not differ from that of *atr* (Fig. 7, A and B). This result showed that crossover remodeling in *atr* is largely independent of MUS81.

Our results showed that crossover redistribution in *atr* is independent of both the Class I and Class II crossover pathways. This finding is consistent with observations made for the sister kinase ATM, in which changes in crossover frequency are also independent of ZMM and MUS81 in Arabidopsis (Kurzbauer et al. 2021). Therefore, we speculate that both ATM and ATR kinases affect meiotic recombination independently of crossover pathway choice.

The Arabidopsis protein FANCD2 was recently shown to promote Class II crossovers in an MUS81-independent manner and to affect Class I crossover distribution (Kurzbauer et al. 2018; Li et al. 2021). We therefore examined the effect of the *fancd2* mutation on the changes in recombination observed for *atr*. We observed a slight though not statistically significant increase in the *420* crossover frequency between *fancd2* and the wild type (Fig. 7C, Supplementary Table S13). Additionally, the frequency in the *atr fancd2* double mutant did not differ from that of the *atr* mutant (Fig. 7C). However, both *fancd2* and *atr* showed significantly lower crossover rates than wild type in the *3.9* interval (Fig. 7D, Supplementary Table S14). The *atr fancd2* double mutant was not different from any of the single mutants. On this basis, we concluded that crossover remodeling in *atr* does not depend on FANCD2 activity. Interestingly, the effects of both mutations do not accumulate in the double mutant. This suggests that the *fancd2* and *atr* mutations lead to similar changes in crossover redistribution, possibly through a common mechanism (resulting in non-additive effects) but are initiated through different pathways (which is why the effects are not interdependent).

It was recently shown that the inactivation of the central element of the synaptonemal complex in *A. thaliana*, through the inactivation of the *ZIPPER1* genes, *ZYP1A* and *ZYP1B*, leads to a similar crossover redistribution as observed in *atr*: the crossover frequency increases in subtelomeric regions and decreases in pericentromeric regions (Capilla-Pérez et al. 2021; France et al. 2021). Therefore, we also examined the effect of combination of the *atr* and *zyp1* mutants for crossover frequency within the *3.9* interval. As expected, both *atr* and *zyp1* exhibited a reduced crossover frequency, with *zyp1* not significantly different from *atr* (Fig. 7E, Supplementary Table S15). However, the *atr zyp1* double mutant showed a lower crossover frequency than either single mutant. This result indicates not only that the effect of *atr* on crossover redistribution is independent of ZYP1, but also that these two mutations act synergistically, mutually enhancing their effects. This suggests that the underlying mechanisms are different in the two mutants.

### *ATR* inactivation affects crossover frequency independently of the pattern of heterozygosity along the chromosome

**Figure 8.**
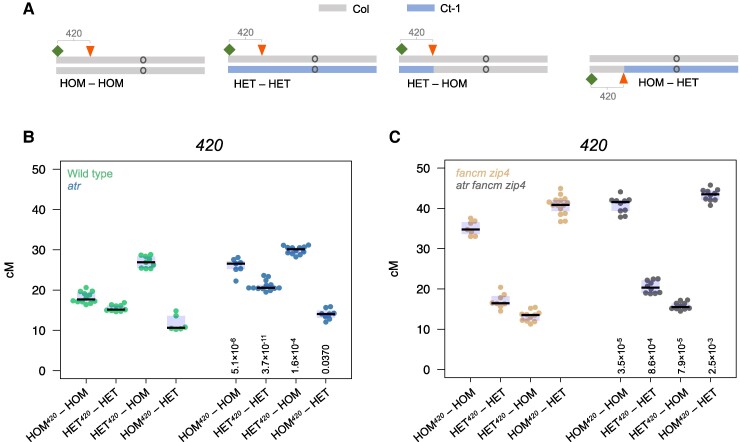
*ATR* inactivation influences crossover frequency independently of the chromosome hetero/homozygosity pattern. **A)** Ideograms of Chromosome 3 in lines differing in heterozygosity pattern. The gray line corresponds to the Col genotype, while the blue line corresponds to the Ct genotype. The locations of the fluorescent reporters defining the *420* intervals are indicated by green square and red arrowheads. **B)***420* crossover frequency (cM) in the HOM*^420^*-HOM, HET*^420^*-HET, HET*^420^*-HOM, and HOM*^420^*-HET genotypes shown in the wild type or *atr*. The center line of a boxplot indicates the median; the upper and lower bounds indicate the 75th and 25th percentiles, respectively. Each dot represents a measurement from one individual. The two-sided *P-*values comparing crossover frequency in *atr* with its wild-type counterpart were estimated by Welch's *t* test and indicated below the boxplots. **C)** As in **B**, but for *fancm zip4* and *atr fancm zip4*.

It is widely accepted that DMC1 recombinase is instrumental in interhomolog bias, stimulating crossover recombination between homologous chromatids (Schwacha and Kleckner 1997; Kurzbauer et al. 2012; Da Ines et al. 2013; Lao et al. 2013). One of the properties that predisposes DMC1 to this role is greater DNA mismatch tolerance during homologous recombination than that of its sister recombinase, RAD51 (Lee et al. 2015, 2017; Luo et al. 2021). In the juxtaposition effect, crossovers are stimulated in heterozygous fragments adjacent to homozygous regions on the same chromosome (Ziolkowski et al. 2015; Blackwell et al. 2020; Dluzewska et al. 2023; Szymanska-Lejman et al. 2023). Therefore, we tested whether impaired DMC1 loading caused by *ATR* inactivation affects this selective crossover targeting in Arabidopsis (Kurzbauer et al. 2012). To this end, we utilized Col/Ct recombinant lines, which exhibit different heterozygosity patterns along Chromosome 3 (Ziolkowski et al. 2015). We analyzed four different combinations of Col/Ct heterozygosity (Fig. 8A):

- “HOM*^420^*-HOM”: Col/Col homozygosity throughout the entire genome
- “HET*^420^*-HET”: Col/Ct heterozygosity throughout the entire genome
- “HET*^420^*-HOM”: Col/Ct heterozygosity in the *420* region, with the rest of Chromosome 3 being Col/Col homozygous
- “HOM*^420^*-HET”: Col/Col homozygosity in the *420* region, with the rest of Chromosome 3 being Col/Ct heterozygous

The *420* index used in the line name indicates the location of the *420* interval in either the homozygous (HOM) or heterozygous (HET) region (Fig. 8A). We measured the crossover frequency in the *420* interval, which overlaps with a region differing in heterozygosity between particular Col/Ct lines. As expected, the HET*^420^*-HOM line exhibited greater crossover than did the HOM*^420^*-HOM line (Welch *t*-test *P* = 9.4 × 10^−11^), while the HOM*^420^*-HET line exhibited a significant reduction in crossover (*P* = 1.3 × 10^−4^). This finding is consistent with heterozygosity stimulating crossover repair when juxtaposed to homozygous regions on the same chromosome (Ziolkowski et al. 2015). Then, we used CRISPR/Cas9 gene editing to knockout *ATR* separately in the four Col/Ct recombinant lines (Supplementary Fig. S4). When we measured the frequency of *420* crossovers in the *atr* background, we observed that all four heterozygosity contexts presented significantly elevated recombination compared with that of the wild type (Fig. 8B, Supplementary Tables S16 and S17). This shows that the heterozygosity juxtaposition effect is preserved in *atr*.

As reported previously, we observed that the *fancm zip4* mutant did not exhibit a heterozygosity juxtaposition effect; instead, the frequency of *420* crossovers is high whenever the *420* interval is homozygous and low whenever it is heterozygous (Fig. 8C) (Ziolkowski et al. 2015; Dluzewska et al. 2023). We decided to determine whether *ATR* inactivation is able to increase the *420* crossover frequency in the Col/Ct recombinant lines with *fancm zip4* in different heterozygosity contexts. Therefore, we crossed the obtained *atr* Col/Ct recombinant lines with their counterparts in the *fancm zip4* background and measured *420* crossovers. For all four tested heterozygosity combinations, *atr fancm zip4* exhibited a significantly greater crossover frequency than *fancm zip4* (Fig. 8C, Supplementary Tables S18 and S19). Taken together, these results suggest that ATR-dependent DMC1 loading does not affect the sensitivity of nucleofilaments toward interhomolog polymorphisms in either the Class I or Class II crossover pathways.

## Discussion

In this study, we present research that expands the body of knowledge on the function of ATR kinase in meiosis, with particular emphasis on crossover recombination. The starting point for our study was an unexpected discovery that ATR inactivation leads to a drastic change in the crossover distribution in Arabidopsis. This change involves an increase in the frequency of recombination in subtelomeric and interstitial chromosome regions at the expense of a decrease in pericentromeric regions. At the same time, the *atr* mutant exhibits full fertility and no visible meiotic chromosome defects, indicating that the redistribution is not a result of DNA repair failure but rather a deregulation of crossover. A similar crossover redistribution profile has been reported for mutants such as *recq4ab*, *fancm* or *sni1*, in which the frequency of Class II crossovers was changed to different degrees and via various mechanisms (Crismani et al. 2012; Girard et al. 2015; Fernandes et al. 2017; Zhu et al. 2021).

To investigate whether this change is also reflected at the recombination hotspot level, we applied our recently developed high-resolution crossover mapping method, *seed-typing* (Szymanska-Lejman et al. 2023). This approach revealed that the crossover topology in the *ChP* interval for the *atr* mutant was similar to that of the wild type, with three hotspots clearly conserved. We observed a reduction in the relative activity of the weakest hotspot, *Nala*, along with a corresponding increase in the activity of the strongest hotspot, *Coco* (Fig. 3). We concluded that although some local changes in hotspot activity are noticeable, the *atr* mutation does not significantly alter the fine-scale distribution of recombination.

Previous studies have shown that in Arabidopsis, ATR controls the loading of DMC1 recombinase onto ssDNA, leading to a drastic increase in the number of DMC1 foci and a moderate decrease in the number of RAD51 foci (Kurzbauer et al. 2012). However, it is not known whether the total number of meiotic DSBs remains unchanged. Our analysis of the γH2A.X focus numbers suggests that the *atr* mutation does not influence DSB abundance (Fig. 4, A and B). This result is consistent with previous observations that the *atr* mutant has a relatively mild phenotype compared with that of the Arabidopsis *atm* mutant (Culligan and Britt 2008). A similar situation may exist in mice, for which no change in DSB number was observed in ATR-deficient lines (Widger et al. 2018). In budding yeast, activation of MEC1 leads to an increase in DSB abundance, but this happens indirectly by extending the duration of prophase I (Gray et al. 2013). Hence, the lack of effect of ATR on DSB numbers appears to be universal across eukaryotes. However, MEC1 was found to be involved in the spatial regulation of DSBs in *trans*, i.e. between homologs (Garcia et al. 2015; Cooper et al. 2016). If plant ATR has a similar function, weakened DSB *trans*-interference in *atr* may result in hyperactivation of strong hotspots and reduced activity of weak hotspots, which is consistent with our observations (Figs. 3 and 4). Regardless of the reason for the change in hotspot activity in *atr*, this property of this mutant is likely responsible for the genome-wide redistribution of crossovers.

Our genome sequencing data indicated that the total number of crossover events increased in *atr* (Fig. 2B). However, this result was not confirmed by the analysis of the number of MLH1 foci, which are broadly recognized as Class I crossover markers (Fig. 4D). Moreover, we did not observe any increase in the frequency of noninterfering Class II crossovers in the *atr zip4* double mutant (Fig. 5, B and C). Hence, the results for MLH1 focus number and chiasma counts/fertility in *atr zip4* appear to contrast with the observations in the genome sequencing analyses. There are several possible explanations for this inconsistency. First, the increases in crossover frequency we observed in genome sequencing data were relatively small and statistically significant for only two chromosomes (Fig. 2B), so it is possible that they remained unnoticeable at the cytological level. Second, sex-averaged crossovers are observed in the genome sequencing approach, while exclusively male-specific recombination is investigated through cytology. Finally, while cytological analyses were performed for Col/Col inbred plants, sequence-based crossover mapping was performed for Col/L*er* hybrids. Col and L*er* differ in terms of the number of alleles for two recombination modifier genes, *HEI10* and *SNI1*, affecting the ZMM pathway and Class II crossovers, respectively (Ziolkowski et al. 2017; Zhu et al. 2021). Consequently, the crossover number in Col/Col is greater than that in Col/L*er* (4.08 vs. 2.79 for female meiosis and 6.13 vs. 5.4 for male meiosis), and substantial differences in the chromosomal distribution of the crossover are also visible (Lian et al. 2022). Therefore, it is possible that the moderate increase in crossovers observed in *atr* Col/L*er* is due to a different combination of crossover modifier alleles than in Col/Col.

Interesting results have been obtained by examining the genetic interaction between *atr* and *fancm* mutants. While both mutations seem to act additively in the subtelomeric interval, causing an independent local increase in crossover frequency (Fig. 6A), their combination leads to increased crossovers above the level observed in both *fancm zip4* and *atr* in the pericentromeric interval (Fig. 6B). These changes occurred independently of the ZMM pathway, indicating that Class II crossovers were redistributed. A potential explanation for this effect is related to the known role of DNA helicases in dissolving recombination intermediates unprotected by ZMM proteins and thus limiting the number of Class II crossovers. In Arabidopsis meiosis, both FANCM and RECQ4 play this role (Crismani et al. 2012; Séguéla-Arnaud et al. 2015). However, while disabling *FANCM* results in an increase in crossover frequency mainly at the ends of chromosomes, the *recq4* mutation triggers a more global increase in pericentromeres (Dluzewska et al. 2023). Although the role of ATR-dependent RECQ4 phosphorylation in meiosis has not been studied, the recruitment of BLM, the human equivalent of RECQ4, is partially dependent on ATR in DNA repair and replication stress (Davies et al. 2004; Rao et al. 2005). Therefore, it is possible that the observed increase in crossover frequency in the pericentromeric regions in *fancm atr* is due to limited recruitment of RECQ4 to these chromosome regions, which in turn triggers repair via Class II crossovers.

Analysis of other genetic interactions, especially with *fancd2* and *zyp1*, provided further insights into the role of ATR in meiotic recombination. The analysis of mutants showed that the effect of *atr* is independent of these proteins. The *atr fancd2* double mutant exhibited a recombination frequency similar to that of each single mutant. On the other hand, *zyp1* acted as an enhancer of *atr* (and *vice versa*), with the crossover frequency in the *3.9* region being lower than in either *zyp1* or *atr* alone. This suggests that while the mechanism of crossover redistribution observed in *atr* may be similar to that in *fancd2*, it differs from the redistribution observed in *zyp1*. This is consistent with the observation that the synaptonemal complex limits Class I crossovers, which increase in *zyp1* (Capilla-Pérez et al. 2021; France et al. 2021) but remain unchanged in *atr* (this work). At the same time, it rules out the possibility that the changes in recombination in *atr* are caused by a potential impact of this kinase on the synaptonemal complex.

DMC1 recombinase is crucial for interhomolog bias by inhibiting the recombination activity of RAD51 and thus limiting sister repair (Schwacha and Kleckner 1997; Cloud et al. 2012; Da Ines et al. 2013, 2022). Unlike RAD51, DMC1 shows a greater tolerance to mismatches arising during strand invasion, which allows DSB repair based on homologous chromosomes (Neale and Keeney 2006). Although Arabidopsis plants with mutations in both genes are sterile, differences in their functionality are manifested by different cytological phenotypes. While *rad51* causes extensive chromosome fragmentation during meiosis due to the inability to repair DSBs, *dmc1* does not cause chromosome damage; although it is unable to repair DSBs using homologous chromatids and hence exhibits no crossovers, it repairs them using sister-chromatids (Couteau et al. 1999; Li et al. 2004; Pradillo et al. 2012). Since DMC1/RAD51 focus ratios increase while the number of DSBs is unaffected in *atr* mutants (Kurzbauer et al. 2012; Fig. 4B), we wondered whether this translates into an enhanced preference for regions with a high level of interhomolog polymorphism. We therefore examined the frequency of crossovers in mosaic lines differing in their heterozygosity pattern (Ziolkowski et al. 2015). With all combinations of hetero/homozygosity, we observed an increase in the *420* crossover frequency whenever ATR was nonfunctional (Fig. 8). Moreover, this effect occurred regardless of whether both Class I and Class II crossovers or only noninterfering Class II crossovers were present (Fig. 8, B and C). We concluded that changes in the loading of recombinases onto ssDNA during nucleofilament formation caused by disabling ATR do not translate into greater recombination activity in heterozygous regions than in homozygous regions and do not contribute to the hetero/homozygosity juxtaposition effect. This finding suggested that the DMC1/RAD51 ratio does not influence crossover/NCO decisions and that the role of these genes is limited to only the strand invasion stage.

In summary, we demonstrate that the effects of ATR on meiotic recombination are not limited only to the initial stages of DSB formation and processing but also translate to the final outcomes of repair. Specifically, we have shown that the loss of ATR leads to a drastic change in crossover distribution along chromosomes, including an increase in crossover frequency within chromosomal arms and a decrease in pericentromeres. This crossover remodeling is not caused by changes in the activity of individual crossover pathways and is independent of several genetic factors that shape recombination in meiosis.

## Materials and methods

### Growth conditions and plant material

Arabidopsis (*Arabidopsis thaliana*) plants were grown in the controlled environment, at 21°C with long day 16/8 h light/dark photoperiods, with 70% humidity and 150 μmol light intensity. To stratify germination seeds were first kept for 48 h in the dark at 4°C.

For measuring recombination frequency fluorescence-tagged lines were used: *420*, kindly provided by Avraham Levy (Melamed-Bessudo et al. 2005), CTLs *1.8*, *1.23*, and *3.9*, kindly provided by Scott Poethig (Wu et al. 2015). T-DNA mutant line *mus81-1* (GABI_113F11) in Col-0 background was obtained from Holger Puchta (Hartung et al. 2006). The Col mutants *fancm-1, zip4-2* and *zyp1-1* were kindly provided by Raphael Mercier (Chelysheva et al. 2007; Crismani et al. 2012; Capilla-Pérez et al. 2021). L*er*-0 and Col/Ct recombinant lines *atr* mutants were generated in this work via CRISPR/Cas9 mutagenesis (see below).

All primer sequences used for genotyping of mutant lines are described in Supplementary Table S20.

### CRISPR/Cas9 mutagenesis of *ATR* in L*er*-0 and in Col/Ct recombinant lines

CRISPR/Cas9 mutagenesis on Arabidopsis plants was performed according to the protocol (Bieluszewski Sura et al. 2022; Bieluszewski Szymanska-Lejman et al. 2022). To obtain *atr* mutant line in L*er*-0 and in Col/Ct juxtaposition lines, a pair of gRNAs targeted within exon 1 of *ATR* were designed (Supplementary Table S21). A following vector for *Agrobacterium* transformation was used: containing the *ATR* gRNA pair under the U3 and U6 promoters, *ICU2::Cas9* transgene, *NapA::dsRED* transgene, and Basta resistance cassette. The pFGC-I2Cas9 binary plasmid was used as the vector for delivering constructs (Bieluszewski Sura et al. 2022; Bieluszewski Szymanska-Lejman et al. 2022). Transformants were selected based on the presence of red fluorescence in seeds or based on resistance to Basta treatment. Selected transformant were genotyped by PCR amplification with primers flanking the *ATR* gRNA target sites (Supplementary Table S21). Sanger sequencing on amplified amplicons was performed to analyze deletion lines. Mutants with heritable deletions causing a shift in the *ATR* open reading frame, and not carrying the CRISPR-Cas9 construct, were identified for further experiments.

### Genotyping-by-sequencing library preparation

DNA from F_2_ plants obtained from Col × L*er* cross was extracted as described (Rowan et al. 2019) and the quality of the DNA was checked in 1% agarose gel. Tagmentation mix was prepared as follows: 2 µL of DNA sample (5 ng/µL concentration), 1 µL of Tagmentation Buffer (40 mm Tris-HCl pH = 7.5, 40 mm MgCl_2_), 0.5 µL of dimethylformamide (DMF), 2.35 µL of Nuclease-free water and 0.05 µL of loaded, in-house produced Tn5 tagmentase. Loading Tn5 with the annealed linker oligonucleotides was previously described (Zhu et al. 2021). The tagmentation step was performed at 55°C for 2 min and then stopped by adding 1 µL 0.1% SDS and incubating at 65°C for 10 min. Amplification of the tagmented DNA was performed using the KAPA2G Robust PCR kit (Sigma) and custom P5 and P7 indexing primers. Each sample was amplified with the unique set of P5 and P7 primers as described (Supplementary Table S22) (Rowan et al. 2019). The quality of libraries was checked on 2% agarose gel, and successful ones were pooled (around 48 libraries in each pool) and size selected—DNA fragments in a range of 400 to 700 bp were excised from 2% agarose gel and extracted using Gel Extraction Kit (A&A Biotechnology). The quality and quantity of the libraries were verified with TapeStation system (Agilent) and Qubit 2.0 fluorometer. Paired-end sequencing of libraries was performed on HiSeq X-10 instrument (Illumina).

### Genotyping-by-sequencing bioinformatics analysis

To identify single-nucleotide polymorphisms (SNPs) within the examined Col × L*er atr* population (220 individuals), demultiplexed paired-end forward and reverse reads were aggregated and aligned to the Col-0 genome reference sequence using BowTie2 (Langmea and Salzberg 2013). The resultant BAM were sorted SAMtools v1.2 (Li and Durbin 2009). The identification of SNPs was carried out with SAMtools and BCFtools (Li 2011). Next, individual sequencing libraries were aligned to the Col-0 genome reference sequence (TAIR10) using default parameters in BowTie2 and cross-referenced with a previously generated SNP list. Following this, the resulting tables of SNPs were filtered to retain only those exhibiting high mapping quality (>100) and high coverage (>2.5×) in R. Libraries with fewer than 95,000 reads were excluded from the analysis. Crossover calling utilized the TIGER pipeline on the filtered files (Rowan et al. 2015). Finally, crossover distribution frequencies were binned into scaled windows and cumulatively aggregated across chromosome arms.

To determine crossover landscape across the *ChP* interval in 142 Col × L*er atr* individuals, the previously described protocol has been used (Szymanska-Lejman et al. 2023). A comprehensive summary of genome sequencing results is provided in Supplementary Table S5. The raw FASTQ data can be found in the GEO under accession number PRJNA1058166.

### *Seed-typing* and recombination frequency measurements in *ChP* and *BT* intervals

Crossover frequency (RF) in *ChP* and *BT* intervals was measured using a seed-based system as previously described (Szymanska-Lejman et al. 2023). RF is expressed as a percentage of recombinants, i.e. seeds that experienced the crossover between two reporter genes, divided by the total number seeds collected from the plant. RF is expressed in cM, followed the formula: RF = 100 × ((NG + NR)/NT), where NG is the number of exclusively green seeds, NR is the number of exclusively red seeds, and NT is the total number of seeds. Images of seeds are captured using a Zeiss epifluorescent stereomicroscope (Discovery V8) at ×6.4 magnification, equipped with a charge-coupled device (CCD; Zeiss Axiocam) camera. The following imaging modes are used: (i) brightfield, (ii) UV illumination with a dsRED filter, and (iii) UV illumination with a GFP filter. The CellProfiler program is used to count the number of seeds based on the protocol outlined in the reference (Kbiri et al. 2022). Due to low recombination rates in such short intervals as *ChP* and *BT*, the identification of single-color recombinants is done manually. Each genotype was analyzed with 5 to 15 biological replicates (i.e. individual plants) grown side by side. Raw seed scoring data are included in Supplementary Tables S6 and S7.

All steps of *seed-typing* method have been described in Szymanska-Lejman et al. (2023). Briefly, *atr* Col-*ChP* × L*er* F_2_ recombinant seeds, single-color ﬂuorescence (-R/– or G-/–) or homozygous for one reporter and hemizygous for the second one (GR/-R or GR/G-), were preselected manually based on seed fluorescence intensity. DNA was extracted using CTAB buffer from the 3-wk-old plants grown from recombinant seeds. Next, DNA was purified using AMPure XP magnetic beads (Beckman-Coulter) and was used as a template for long-range PCR. Amplification of *ChP* interval (26.3 kb) was divided into three separate reactions using primers listed in Supplementary Table S23. The reaction was performed in 15 µL and contained 0.2 to 10 ng of template DNA, 1.2 µL of 2.5 mmol/L primers, 3 µL of buffer, 1.2 µL of dNTP and 0.3 µL of Polymerase (PrimeSTAR GXL Polymerase, TaKaRa Bio, Shiga, Japan). The efficiency of the amplification (30 cycles 98°C 10 s, 68°C 12 min) was verified in 1% agarose gel, and amplicons generated from the same DNA sample were pooled, purified, and eluted (Clean-Up Concentrator, A&A Biotechnology). These pooled PCR products were subjected to tagmentation to prepare libraries for sequencing as described in: Genotyping-by-sequencing library preparation. Libraries were visualized to ensure comparable fragment intensity and pooled. Concentrated libraries were subjected to size selection in 2% gel. Library quality and quantity were verified, followed by paired-end sequencing on the HiSeq X−10 instrument (Illumina) with 576 libraries per lane. Paired-end sequencing was performed on HiSeq X−10 instrument (Illumina).

### Crossover frequency measurement using FTL seed-based system

Crossover rate measurements using seed-based system were performed as described previously (Ziolkowski et al. 2015; Kbiri et al. 2022). Pictures of seeds were acquired as described in the section “Seed-typing and recombination frequency measurements in *ChP* and *BT* intervals”. The images were processed by CellProfiler software (Carpenter et al. 2006), which identified seed boundaries and assigned a dsRed and eGFP fluorescence intensity values to each seed object. Thresholds between fluorescent and nonfluorescent seeds were set manually using fluorescence histograms for each color. Crossover frequency was calculated as cM = 100 × (1–(1–2(NG + NR)/NT)/2), where NG is the number of green alone seeds, NR is the number of red alone seeds, and NT is the total number of seeds. Each genotype was analyzed with 5 to 17 biological replicates (i.e. individual plants), for which the crossover rate was measured based on the segregation of reporters in seeds. Comparisons between genotypes were carried out by growing all the plants side by side.

### Fertility assays

Seed set was assessed from five fruits, located at positions 6 through 10 of the main stem, in seven plants per genotype. Each biological replicate corresponded to one plant.

### Cytogenetic techniques

For DAPI spreading of pollen mother cells, fixed flower buds were used in Carnoy's solution (6:3:1, ethanol: chloroform: acetic acid) according to the procedure described by Ross et al. (1996). For immunostaining of meiotic proteins, the spreading technique described by Armstrong et al. (2009) was performed on fresh material. The following primary antibodies were used: α-γH2AX (mouse, 1:500, #05-636, Upstate), α-ASY1 (rabbit, 1:500), α-MLH1 (rabbit, 1:300), and α-ZYP1 (rat, 1:500). These antibodies (except α-γH2AX) were kindly provided by Prof. Chris Franklin (University of Birmingham, UK). The following secondary antibodies were used: α-mouse (Cy3, 1:100, BioLegend), α-rabbit (FITC, 1:100, Agrisera), α-rabbit (Alexa Fluor 555, 1:500, Molecular Probes), and α-rat (FITC, 1:100, Agrisera). Slides were imaged using an Olympus BX61 epifluorescence microscope equipped with an Olympus DP71 CCD digital camera. The following laser lines and filter sets were used: For fluorescein (FITC), a 488 nm laser line with a 530/40 filter set was applied, with excitation at 498 nm and emission at 517 nm. For cyanine (Cy3), the same 488 nm laser line was used with a 593/40 filter set, providing excitation at 554 nm and emission at 566 nm. For Alexa Fluor 555, a 488 nm laser line with a 555/20 filter set was employed, with excitation at 553 nm and emission at 568 nm. For foci number comparison one-way ANOVA with Tukey HSD was used to test for statistical significance.

### Statistical analyses

Statistical analyses were performed using calculators from Statistics Kingdom (http://www.statskingdom.com) using a significance level (α) of 0.05. Details of all statistical tests are provided in the figure legends, and the complete statistical data are available in Supplementary Data Set 1.

### Accession numbers

Genes described in this article can be found in the TAIR database (https://www.arabidopsis.org) under the following accession numbers: *ATR* (AT5G40820); *MUS81* (AT4G30870); *ZIP4* (AT1G56590); *FANCM* (AT1G35530); *FANCD2* (AT4G14970); *SNI1* (AT4G18470); *SPO11-1* (At3g13170); *ZYP1A* (AT1G22260); *ZYP1B* (AT1G22275).

## Supplementary Material

koae292_Supplementary_Data

## Data Availability

The data underlying this article are available in the article and in its online supplementary material. The raw FASTQ data can be found in the GEO under accession number PRJNA1058166.

## Supplementary data

The following materials are available in the online version of this article.

**Supplementary Figure S1.** ATR CRISPR/Cas9-mediated mutagenesis in L*er* accession.

**Supplementary Figure S2.** Comparison of crossover numbers between wild type and the *atr* mutant.

**Supplementary Figure S3.** Cytological characterization of the *atr* mutant.

**Supplementary Figure S4.** *ATR* CRISPR/Cas9-mediated mutagenesis in Col/Ct recombinant lines.

**Supplementary Table S1.** *420* fluorescent seed count data for the wild type, *atr*, *sni1,* and *atr sni1* double mutants in Col/Col inbred lines.

**Supplementary Table S2.** *3.9* fluorescent seed count data for the wild type, *atr*, *sni1,* and *atr sni1* double mutants in Col/Col inbred lines.

**Supplementary Table S3.** *CTL 1.18* and *1.23* fluorescent seed count data for the wild type and *atr* mutant in Col/Col inbred lines.

**Supplementary Table S4.** *420* and *3.9* fluorescent seed count data for the wild type and *atr* mutant in Col/L*er* hybrid lines.

**Supplementary Table S5.** Summary of genome sequencing of atr Col × L*er* F_2_, wild-type Col × L*er* F_2_, and Col-*ChP* × L*er* F_2_ (seed-typing).

**Supplementary Table S6.** *ChP* and *BT* fluorescent seed count data for the wild type and *atr* mutant in Col/Col inbred lines.

**Supplementary Table S7.** *ChP* and *BT* fluorescent seed count data for the wild type and *atr* mutant in Col/L*er* hybrid lines.

**Supplementary Table S8.** Seed set per silique in Col in the wild type, *atr*, *zip4,* and *atr zip4* mutant lines.

**Supplementary Table S9.** *420* fluorescent seed count data for the wild type, *atr*, *fancm* single mutants, *atr fancm*, *fancm zip4* double mutants, and *atr fancm zip4* triple mutants in Col/Col inbred lines.

**Supplementary Table S10.** *3.9* fluorescent seed count data for the wild type, *atr*, *fancm zip4,* and *atr fancm zip4* mutants in Col/Col inbred lines.

**Supplementary Table S11.** *420* fluorescent seed count data for the wild type, *atr*, *mus81,* and *atr mus81* double mutants in Col/Col inbred lines.

**Supplementary Table S12.** *3.9* fluorescent seed count data for the wild type, *atr*, *mus81,* and *atr mus81* double mutants in Col/Col inbred lines.

**Supplementary Table S13.** *420* fluorescent seed count data for the wild type, *atr*, *fancd2,* and *atr fancd2* double mutants in Col/Col inbred lines.

**Supplementary Table S14.** *3.9* fluorescent seed count data for the wild type, *atr*, *fancd2,* and *atr fancd2* double mutants in Col/Col inbred lines.

**Supplementary Table S15.** 3.9 fluorescent seed count data for the wild type, *atr*, *zyp1,* and *atr zyp1* double mutants in Col/Col inbred lines.

**Supplementary Table S16.** *420* fluorescent seed count data for the wild type in recombinant lines with differing pattern of heterozygosity.

**Supplementary Table S17.** *420* fluorescent seed count data for the *atr* single mutant in Col/Ct recombinant lines with differing patterns of heterozygosity.

**Supplementary Table S18.** *420* fluorescent seed count data for the *fancm zip4* double mutant in Col/Ct recombinant lines with differing patterns of heterozygosity.

**Supplementary Table S19.** *420* fluorescent seed count data for the *atr fancm zip4* triple mutant in Col/Ct recombinant lines with differing patterns of heterozygosity.

**Supplementary Table S20.** Primers used for mutant genotyping.

**Supplementary Table S21.** Primers used for ATR targeting in Ct and L*er* (gRNA) and *atr* CRISPR/Cas9 mutant Sanger sequencing and genotyping.

**Supplementary Table S22.** Primers used for genome sequencing and seed-typing library preparation.

**Supplementary Table S23.** Primers used for LR-PCR in seed-typing.

**Supplementary Data Set S1.** Statistical data.

## Dive Curated Terms

The following phenotypic, genotypic, and functional terms are of significance to the work described in this paper:

RPA Gramene: AT2G35210
RPA Araport: AT2G35210

ATM Gramene: AT3G48190
ATM Araport: AT3G48190

MUS81 Gramene: AT4G30870
MUS81 Araport: AT4G30870

ZYP1A Gramene: AT1G22260
ZYP1A Araport: AT1G22260

ZYP1B Gramene: AT1G22275
ZYP1B Araport: AT1G22275

MSH4 Gramene: AT4G17380
MSH4 Araport: AT4G17380

ZIP4 Gramene: AT1G56590
ZIP4 Araport: AT1G56590

DMC1 Gramene: AT3G22880
DMC1 Araport: AT3G22880

ZYP1 Gramene: AT1G22275; AT1G22260
ZYP1 Araport: AT1G22275; AT1G22260

RAD51 Gramene: AT5G20850
RAD51 Araport: AT5G20850

SNI1 Gramene: AT4G18470
SNI1 Araport: AT4G18470

ATR Gramene: AT5G40820
ATR Araport: AT5G40820

FANCD2 Gramene: AT4G14970
FANCD2 Araport: AT4G14970
