# Supplementary material for: The kinase ATR controls meiotic crossover distribution at the genome scale in Arabidopsis
Source: Plant Cell. 2024 Oct 29;37(1):koae292. doi: 10.1093/plcell/koae292 (PMC11663591; doi:10.1093/plcell/koae292)
Supplement: koae292_Supplementary_Data [file koae292_supplementary_data.zip › ATR supplement v.2.pdf]

The kinase ATR controls meiotic crossover distribution at the genome scale in Arabidopsis

Longfei Zhu, Julia Dluzewska, Nadia Fernández-Jiménez, Rajeev Ranjan, Alexandre Pelé, Wojciech Dziegielewski, Maja Szymanska-Lejman, Karolina Hus, Julia Górna, Mónica Pradillo and Piotr A. Ziolkowski

SUPPLEMENTARY DATA

Supplementary Figures

A

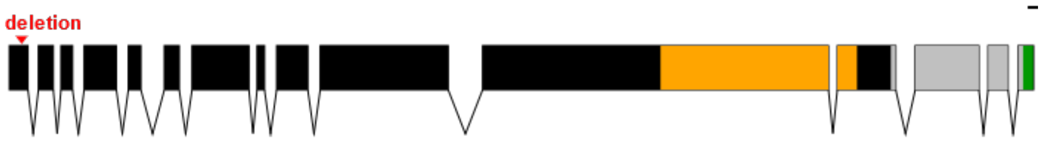

B

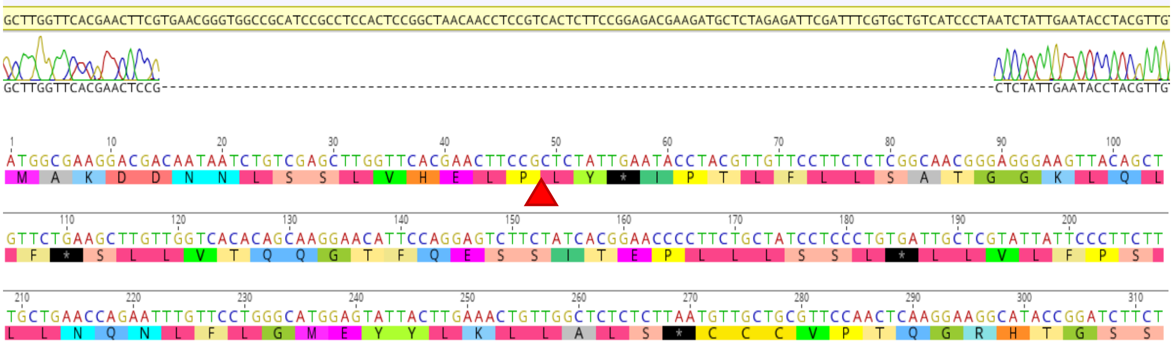

Supplementary Figure S1. ATR CRISPR/Cas9-mediated mutagenesis in L*er* accession. A, Gene model for *A. thaliana ATR*. The CRISPR-Cas9-induced deletion site is indicated by red arrowhead. Rectangles represent exons while connecting lines represent introns. Location of sequences encoding functional domains are indicated by colors: orange for FAT domain, gray for PI3K/PI4K catalytic domain and green for FATC domain. Native ATR protein constitutes of 2702 amino acid residues. Scale bar, 100 bp. B, *ATR* coding sequence used as reference shown with yellow background. Mutagenesis by CRISPR/Cas9 resulted in 101 bp deletion within the first exon, visualized by Sanger sequencing. Red triangle indicates deletion site, which causes a frame shift and formation of multiple pre-mature STOP codons (shown as black squares). Supports Figure 1F.

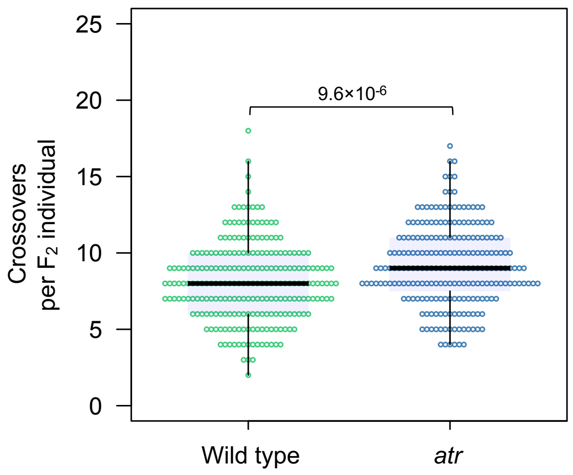

Supplementary Figure S2. Comparison of crossover numbers between wild type and the *atr* mutant. Each dot represents a single F_2 individual. For wild type, 200 randomly selected individuals were plotted. Significance was assessed by Mann-Whitney U test. The center line of a boxplot indicates the median; the upper and lower bounds indicate the 75th and 25th percentiles, respectively; the error bars indicate the minimum and maximum. Supports Figure 2B.

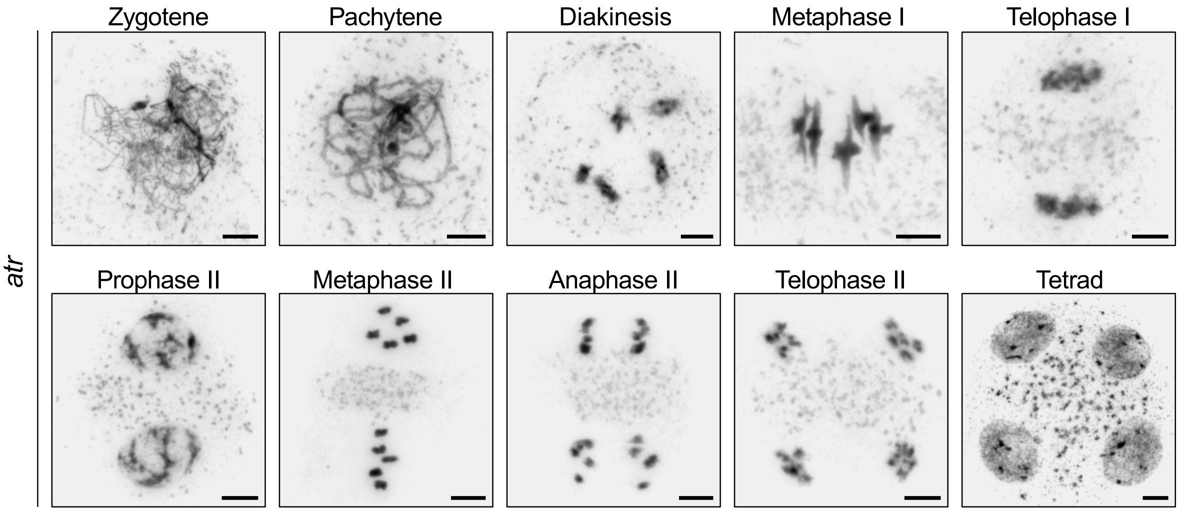

Supplementary Figure S3. Cytological characterization of the *atr* mutant. The stages of meiotic progression were labeled. 134 cells in the first meiotic division and 177 in the second meiotic division from three different plants were quantified. Scale bar, 5 µm. Supports Figure 4A.

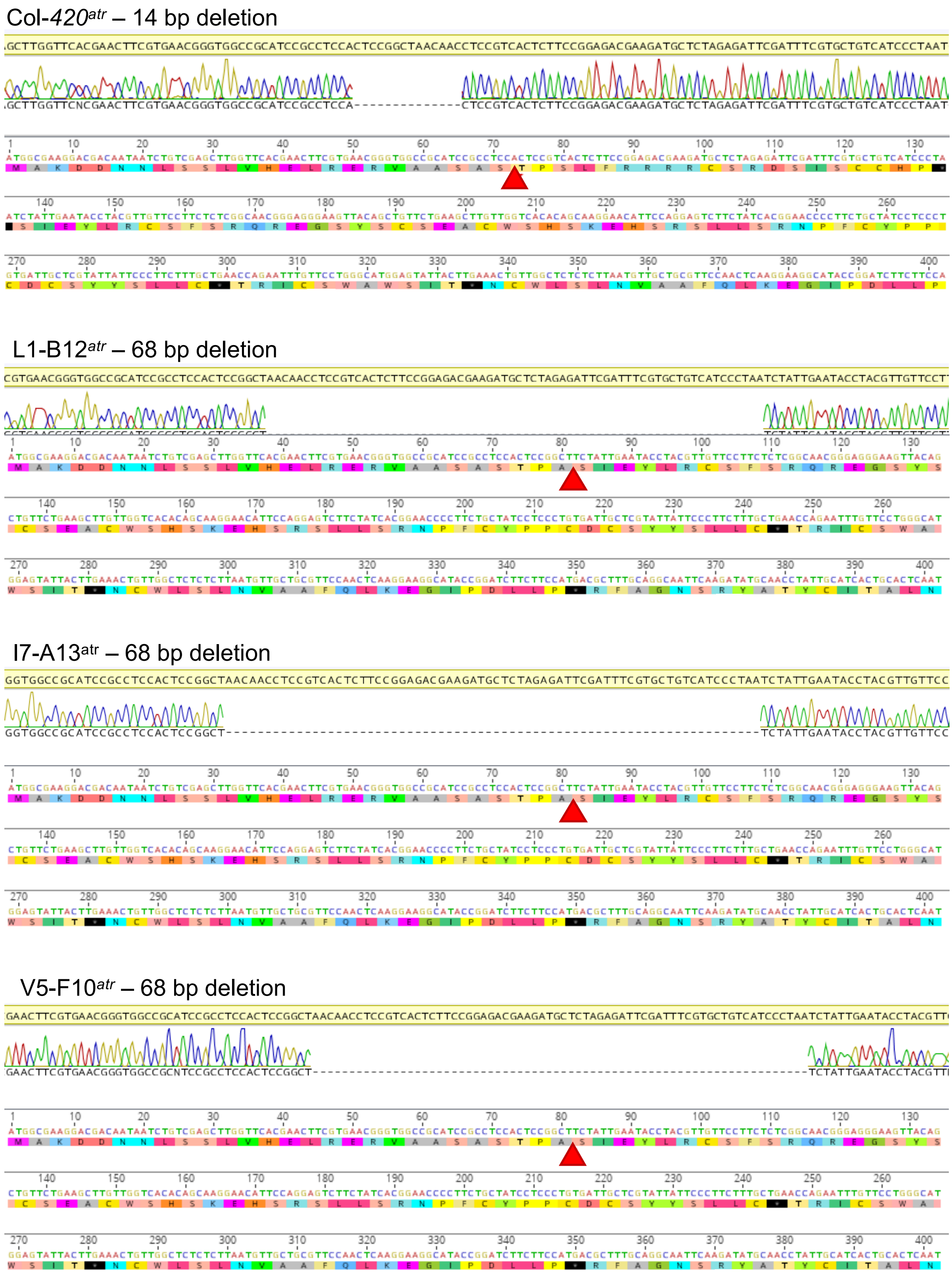

Supplementary Figure S4. *ATR* CRISPR/Cas9-mediated mutagenesis in Col/Ct recombinant lines. *ATR* coding sequence used as reference shown with yellow background. Mutagenesis by CRISPR/Cas9 resulted in a deletion within the first exon of *ATR,* visualized by Sanger sequencing: 14 bp deletion in Col-*420* line and 68 bp deletion in L1-B12 (HET-HET variant),

I7-A13 (HET-HOM variant), V5-F10 (HOM-HET variant). Red triangle indicates deletion site, which causes a frame shift and formation of multiple pre-mature STOP codons (shown as black squares). See Supplementary Figure 2 for the location of the deletion site in relation to the gene model. Supports Figure 8.

Supplementary Tables

Supplementary Table S1. *420* fluorescent seed count data for the wild type, *atr*, *sni1* and *atr sni1* double mutants in Col/Col inbred lines.

Genotype	Total	Green alone	Red alone	Red+Green	Non-colour	cM
Wild type	2413	188	212	1637	376	18.24
Wild type	2383	196	198	1578	411	18.19
Wild type	2432	196	207	1603	426	18.23
Wild type	2416	218	220	1580	398	20.16
Wild type	2452	221	240	1639	352	21.01
Wild type	2198	220	180	1457	341	20.25
Wild type	2144	189	190	1399	366	19.60
Wild type	2442	235	237	1545	425	21.68
atr	2421	257	254	1556	354	23.98
atr	1944	184	220	1251	289	23.56
atr	2241	227	236	1443	335	23.40
atr	2322	252	237	1532	301	23.92
atr	2008	209	224	1301	274	24.59
atr	1988	220	206	1272	290	24.41
atr	1053	111	106	676	160	23.33
atr	2347	251	262	1490	344	24.98
atr	1793	209	188	1137	259	25.36
sni1	822	98	106	503	115	29.03
sni1	916	142	95	580	99	30.54
sni1	1262	148	143	795	176	26.60
sni1	886	113	98	572	103	27.63
sni1	1231	146	159	778	148	28.97
sni1	1782	228	200	1141	213	27.91
sni1	902	108	133	559	102	31.76
atr sni1	487	75	71	293	48	36.72
atr sni1	1741	249	248	1032	212	34.50
atr sni1	1576	254	259	912	151	40.93
atr sni1	1516	227	213	889	187	35.23
atr sni1	1025	149	143	639	94	34.41
atr sni1	1068	164	180	622	102	40.35
atr sni1	862	136	136	521	69	39.26
atr sni1	830	127	121	495	87	36.56

Supplementary Table S2. *3.9* fluorescent seed count data for the wild type, *atr*, *sni1* and *atr sni1* double mutants in Col/Col inbred lines.

Genotype	Total	Green alone	Red alone	Red+Green	Non-colour	cM
Wild type	2423	208	171	1657	387	17.10
Wild type	1315	107	97	902	209	16.95
Wild type	2379	192	187	1609	391	17.45
Wild type	2399	205	205	1569	420	18.87
Wild type	2386	170	190	1642	384	16.44
Wild type	2282	173	210	1516	383	18.49
Wild type	2440	218	191	1629	402	18.47
Wild type	2423	208	171	1657	387	17.10
Wild type	1315	107	97	902	209	16.95
atr	2276	167	132	1548	429	14.14
atr	2293	134	169	1545	445	14.23
atr	1840	109	133	1225	373	14.15
atr	2460	174	151	1701	434	14.22
atr	1453	95	83	1004	271	13.11
atr	2221	139	148	1532	402	13.89
atr	1890	156	104	1301	329	14.86
atr	2356	177	148	1636	395	14.91
atr	2207	147	135	1470	455	13.72
atr	2253	157	140	1520	436	14.19
sni1	869	52	54	603	160	13.05
sni1	892	62	47	615	168	13.07
sni1	343	21	20	233	69	12.77
sni1	424	29	21	292	82	12.58
sni1	880	52	49	614	165	12.22
sni1	715	47	48	481	139	14.31
atr sni1	345	30	16	240	59	14.37
atr sni1	415	22	30	290	73	13.43
atr sni1	751	45	42	524	140	12.35
atr sni1	590	41	31	403	115	13.06
atr sni1	527	30	40	357	100	14.31
atr sni1	497	29	37	346	85	14.30
atr sni1	509	40	29	354	86	14.63
atr sni1	383	28	19	260	76	13.13
atr sni1	768	59	40	520	149	13.85
atr sni1	467	35	26	323	83	14.05

Supplementary Table S3. CTL *1.18* and *1.23* fluorescent seed count data for the wild type and *atr* mutant in Col/Col inbred lines.

Interval	Genotype	Total	Green alone	Red alone	Red+Green	Non-colour	cM
1.18	Wild type	1229	118	93	831	187	18.97
1.18	Wild type	1305	115	99	874	217	18.02
1.18	Wild type	1406	127	111	941	227	18.67
1.18	Wild type	1445	149	106	960	230	19.56
1.18	Wild type	1063	104	87	705	167	19.96
1.18	Wild type	1167	88	118	767	194	19.57
1.18	Wild type	708	62	66	461	119	20.10
1.18	*atr*	1208	124	123	786	175	23.12
1.18	*atr*	1604	159	158	1044	243	22.24
1.18	*atr*	795	85	65	527	118	21.09
1.18	*atr*	756	69	91	492	104	24.06
1.18	*atr*	992	103	107	611	171	24.06
1.18	*atr*	1611	149	166	1057	239	21.97
1.18	*atr*	1213	125	121	778	189	22.90
1.18	*atr*	1208	124	123	786	175	22.78
1.23	Wild type	1338	85	94	904	255	14.42
1.23	Wild type	1283	91	65	897	230	13.00
1.23	Wild type	1030	61	60	727	182	12.48
1.23	Wild type	1256	59	88	872	237	13.36
1.23	Wild type	1051	71	60	723	197	12.53
1.23	Wild type	1088	72	60	759	197	12.97
1.23	Wild type	1115	76	64	786	189	13.46
1.23	Wild type	1660	107	95	1127	331	13.02
1.23	*atr*	1463	120	100	1012	231	16.38
1.23	*atr*	1195	104	86	798	207	17.42
1.23	*atr*	1326	108	117	893	208	18.72
1.23	*atr*	1463	120	100	1012	231	16.38
1.23	*atr*	1355	89	94	936	236	14.57
1.23	*atr*	922	80	77	606	159	18.79
1.23	*atr*	1195	117	66	817	195	16.71

Supplementary Table S4. *420* and *3.9* fluorescent seed count data for the wild type and *atr* mutant in Col/L*er* hybrid lines.

Interval	Genotype	Total	Green alone	Red alone	Red+Green	Non-colour	cM
420	Wild type	2311	154	160	1614	383	14.66
420	Wild type	2072	148	159	1407	358	16.12
420	Wild type	2263	177	149	1555	382	15.63
420	Wild type	2045	148	149	1377	371	15.77
420	Wild type	1679	115	110	1167	287	14.44
420	Wild type	2149	167	145	1488	349	15.76
420	Wild type	1986	124	133	1395	334	13.91
420	*atr*	2150	211	202	1418	319	21.53
420	*atr*	1984	187	203	1309	285	22.10
420	*atr*	2249	221	200	1505	323	20.90
420	*atr*	2026	223	170	1335	298	21.77
420	*atr*	2115	224	195	1387	309	22.30
420	*atr*	1791	142	189	1176	284	20.60
420	*atr*	1878	194	204	1229	251	24.10
420	*atr*	1479	159	147	961	212	23.44
420	*atr*	1627	152	171	1057	247	22.35
420	*atr*	2372	225	219	1557	371	20.90
420	*atr*	2037	235	200	1297	305	24.31
420	*atr*	1721	172	152	1132	265	21.04
420	*atr*	2339	275	190	1535	339	22.39
3.9	Wild type	2264	209	173	1521	361	18.60
3.9	Wild type	1474	128	111	975	260	17.80
3.9	Wild type	2053	191	189	1363	310	20.64
3.9	Wild type	934	98	72	613	151	20.25
3.9	Wild type	1903	179	171	1227	326	20.49
3.9	Wild type	2159	215	173	1403	368	19.96
3.9	Wild type	1681	177	124	1083	297	19.88
3.9	*atr*	2280	183	124	1560	413	14.52
3.9	*atr*	2098	131	148	1431	419	13.44
3.9	*atr*	2157	149	136	1465	407	14.22
3.9	*atr*	2144	116	126	1481	421	12.01
3.9	*atr*	2378	197	136	1617	428	15.15
3.9	*atr*	2251	158	139	1556	398	14.20
3.9	*atr*	2176	159	143	1480	394	15.00

Supplementary Table S5. Summary of genome sequencing of *atr* Col × L*er* F_2, wild-type Col × L*er* F_2 and Col-*ChP* × L*er* F_2 (*seed-typing*).

Experiment	Total crossovers	Total reads	Number of individuals	Average reads per library	Reads per SNP
atr Col × L*er* F_2	2,016	145,212,636	220	660,057	296
wild-type Col × L*er* F_2	1,922	45,676,147	238	191,916	93
Seed-typing	142	37,541,621	142	264,377	151,377

Supplementary Table S6. *ChP* and *BT* fluorescent seed count data for the wild type (WT) and *atr* mutant in Col/Col inbred lines.

Genotype	Sample ID	Number of red recombinants	Number of green recombinants	Total number of recombinants	Total number of seeds	RF (cM)
inbred-ChP-WT	1	4	7	11	3424	0.321
	2	11	8	19	6286	0.302
	3	5	8	13	4508	0.288
	4	8	7	15	6637	0.226
	5	4	6	10	3965	0.252
inbred-ChP-atr	1	11	11	22	7411	0.297
	2	2	3	5	5130	0.097
	3	10	6	16	2489	0.643
	4	6	7	13	5054	0.257
	5	5	3	8	4357	0.184
	6	8	10	18	4720	0.381
	7	5	7	12	4304	0.279
	8	4	5	9	1850	0.486
	9	10	7	17	4923	0.345
	10	3	3	6	3590	0.167
	11	4	4	8	5854	0.137
	12	6	9	15	6572	0.228
	13	8	8	16	5663	0.283
inbred-BT-WT	1	5	7	12	4152	0.289
	2	5	2	7	4257	0.164
	3	4	2	6	4596	0.131
	4	4	11	15	6243	0.240
	5	9	2	11	4611	0.239
inbred-BT-atr	1	1	1	2	2188	0.091
	2	5	5	10	3120	0.321
	3	1	1	2	4184	0.048
	4	3	4	7	3788	0.185
	5	3	5	8	4279	0.187
	6	2	2	4	2707	0.148
	7	5	4	9	5379	0.167

	8	10	4	14	6326	0.221
	9	8	5	13	4884	0.266
	10	3	2	5	2200	0.227
	11	4	4	8	3134	0.255
	12	2	5	7	4359	0.161

Supplementary Table S7. *ChP* and *BT* fluorescent seed count data for the wild type (WT) and *atr* mutant in Col/L*er* hybrid lines.

Genotype	Sample ID	Number of red recombinants	Number of green recombinants	Total number of recombinants	Total number of seeds	RF (cM)
hybrid-ChP-WT	1	17	5	22	5303	0.415
	2	6	3	9	1862	0.483
	3	17	29	46	9916	0.464
	4	8	9	17	3486	0.488
	5	9	10	19	4051	0.469
	6	4	3	7	1876	0.373
hybrid-ChP-atr	1	10	11	21	6876	0.305
	2	8	5	13	6308	0.206
	3	4	4	8	3046	0.263
	4	3	3	6	2601	0.231
	5	2	4	6	3599	0.167
	6	6	4	10	4561	0.219
	7	3	5	8	3290	0.243
	8	13	13	26	9054	0.287
	9	21	15	36	10674	0.337
	10	8	12	20	8537	0.234
hybrid-BT-WT	1	5	5	10	8119	0.123
	2	3	3	6	8450	0.071
	3	5	9	14	4478	0.313
	4	2	1	3	3822	0.078
	5	4	6	10	6403	0.156
	6	2	1	3	2001	0.150
	7	3	3	6	2987	0.201
	8	2	2	4	4051	0.099
	9	1	3	4	2487	0.161
	10	1	2	2	4590	0.044
	11	6	5	11	5581	0.197
hybrid-BT-atr	1	3	5	8	5763	0.139
	2	5	5	10	5878	0.170
	3	5	2	7	5957	0.118

	4	2	4	6	3082	0.195
	5	3	5	8	5916	0.136

Supplementary Table S8. Seed set per silique in Col in the wild type, *atr*, *zip4* and *atr zip4* mutant lines. The number of seeds was counted manually in 7-8 plants per genotype and 5 fruits per plant, located at positions 6 through 10 of the main stem. Mean values per individual are showed.

		Seeds per silique in Col, plant #							
**Genotype**	**Silique**	**1**	**2**	**3**	**4**	**5**	**6**	**7**	**8**
**Wild type**	**1**	50	56	52	46	54	40	56	
	**2**	52	56	46	52	57	54	54	
	**3**	54	52	53	53	42	42	40	
	**4**	51	49	54	48	47	56	50	
	**5**	50	56	52	59	50	54	43	
	**Mean**	**51.4**	**53.8**	**51.4**	**51.6**	**50**	**49.2**	**48.6**	
atr	**1**	55	46	58	50	52	44	54	48
	**2**	54	50	56	49	54	53	46	51
	**3**	46	54	50	53	40	52	56	50
	**4**	50	52	40	52	46	42	54	45
	**5**	47	46	48	51	57	50	58	58
	**Mean**	**50.4**	**49.6**	**50.4**	**51**	**49.8**	**48.2**	**53.6**	**50.4**
zip4	**1**	2	0	3	4	7	5	4	
	**2**	5	4	1	4	4	0	5	
	**3**	5	2	3	2	6	4	6	
	**4**	5	1	0	2	2	4	5	
	**5**	6	4	1	2	1	0	2	
	**Mean**	**4.6**	**2.2**	**1.6**	**2.8**	**4**	**2.6**	**4.4**	
atr zip4	**1**	4	2	4	4	3	3	5	
	**2**	4	2	2	0	2	1	4	
	**3**	4	3	5	4	3	2	5	
	**4**	2	0	3	4	0	3	0	
	**5**	2	2	4	1	1	3	3	
	**Mean**	**3.2**	**1.8**	**3.6**	**2.6**	**1.8**	**2.4**	**3.4**	

Supplementary Table S9. *420* fluorescent seed count data for the wild type, *atr*, *fancm* single mutants, *atr fancm*, *fancm zip4* double mutants and *atr fancm zip4* triple mutants in Col/Col inbred lines.

Genotype	Total	Green alone	Red alone	Red+Green	Non-colour	cM
Wild type	815	79	82	522	132	22.22
Wild type	967	101	95	638	133	22.89
Wild type	1048	121	93	690	144	23.08
Wild type	1374	163	121	874	216	23.41
Wild type	1610	173	139	1037	261	21.74
Wild type	970	102	89	638	141	22.14
Wild type	669	65	68	433	103	22.39
atr	1171	146	140	710	175	28.48
atr	1119	153	127	693	146	29.32
atr	1002	111	137	630	124	28.94
atr	1330	183	163	810	174	30.74
atr	985	127	128	612	118	30.56
atr	1355	185	148	852	170	28.69
atr	789	85	94	510	100	26.09
fancm	1166	257	240	626	43	38.41
fancm	1746	297	253	1026	170	39.17
fancm	889	142	130	518	99	37.70
fancm	1150	159	186	696	109	36.75
fancm	949	210	189	509	41	39.89
fancm	941	146	141	556	98	37.55
fancm	1532	322	340	816	54	36.85
fancm	1127	155	181	675	116	36.46
fancm	893	140	129	537	87	36.95
fancm	961	159	137	577	88	38.03
fancm	844	124	119	515	86	34.87
atr fancm	911	158	152	527	74	43.48
atr fancm	1722	317	273	978	154	43.90
atr fancm	1722	318	273	977	154	44.00
atr fancm	1373	259	230	778	106	46.36
atr fancm	1260	241	206	708	105	46.10
atr fancm	1330	241	198	774	117	41.70
atr fancm	1500	278	237	884	101	44.02
atr fancm	1035	193	182	573	87	47.53
atr fancm	809	168	161	446	34	43.20
atr fancm	1477	266	245	862	104	44.50
fancm zip4	2293	348	332	1391	222	36.21

fancm zip4	2145	327	282	1320	216	34.26
fancm zip4	2023	310	314	1185	214	38.11
fancm zip4	2145	327	282	1320	216	34.26
fancm zip4	2040	440	450	1079	71	35.70
fancm zip4	2036	323	287	1198	228	36.69
fancm zip4	2224	346	337	1305	236	37.89
fancm zip4	2339	380	364	1400	195	39.68
fancm zip4	1570	356	331	834	49	35.33
atr fancm zip4	1856	347	300	1086	123	44.97
atr fancm zip4	1392	234	241	808	109	43.65
atr fancm zip4	2031	357	351	1182	141	44.97
atr fancm zip4	1055	209	164	604	78	45.88
atr fancm zip4	1602	297	276	903	126	46.65
atr fancm zip4	1591	292	265	925	109	45.24
atr fancm zip4	2224	402	374	1267	181	45.03
atr fancm zip4	989	187	165	557	80	46.32
atr fancm zip4	1612	306	259	917	130	45.32
atr fancm zip4	853	190	160	468	35	42.35
atr fancm zip4	786	140	122	457	67	42.26
atr fancm zip4	1437	247	261	795	134	45.87

Supplementary Table S10. *3.9* fluorescent seed count data for the wild type, *atr*, *fancm zip4* and *atr fancm zip4* mutants in Col/Col inbred lines.

Genotype	Total	Green alone	Red alone	Red+Green	Non-colour	cM
Wild type	2423	208	171	1657	387	17.10
Wild type	1315	107	97	902	209	16.95
Wild type	2379	192	187	1609	391	17.45
Wild type	2399	205	205	1569	420	18.87
Wild type	2386	170	190	1642	384	16.44
Wild type	2282	173	210	1516	383	18.49
Wild type	2440	218	191	1629	402	18.47
Wild type	2423	208	171	1657	387	17.10
Wild type	1315	107	97	902	209	16.95
atr	2276	167	132	1548	429	14.14
atr	2293	134	169	1545	445	14.23
atr	1840	109	133	1225	373	14.15
atr	2460	174	151	1701	434	14.22
atr	1453	95	83	1004	271	13.11
atr	2221	139	148	1532	402	13.89
atr	1890	156	104	1301	329	14.86
atr	2356	177	148	1636	395	14.91
atr	2207	147	135	1470	455	13.72
atr	2253	157	140	1520	436	14.19
fancm zip4	2113	122	99	1455	437	11.07
fancm zip4	2377	131	137	1614	495	11.99
fancm zip4	2295	125	124	1552	494	11.51
fancm zip4	2384	146	133	1613	492	12.48
fancm zip4	2483	127	136	1682	538	11.22
fancm zip4	2141	111	142	1455	433	12.61
atr fancm zip4	1632	129	115	1080	308	16.28
atr fancm zip4	1900	132	132	1277	359	15.02
atr fancm zip4	1641	124	101	1099	317	14.81
atr fancm zip4	1253	93	98	848	214	16.63
atr fancm zip4	1570	112	101	1059	298	14.64
atr fancm zip4	1458	84	115	998	261	14.73
atr fancm zip4	1717	98	129	1187	303	14.23
atr fancm zip4	1603	108	126	1065	304	15.85
atr fancm zip4	1661	105	128	1130	298	15.18

Supplementary Table S11. *420* fluorescent seed count data for the wild type, *atr*, *mus81* and *atr mus81* double mutants in Col/Col inbred lines.

Genotype	Total	Green alone	Red alone	Red+Green	Non-colour	cM
Wild type	1179	136	109	780	154	23.55
Wild type	1761	194	164	1164	239	22.97
Wild type	1827	215	165	1182	265	23.58
Wild type	704	79	65	448	112	23.13
Wild type	631	55	63	421	92	20.88
Wild type	665	76	60	423	106	23.12
Wild type	1179	136	109	780	154	25.87
atr	1124	240	281	589	14	27.01
atr	2049	239	242	1302	266	27.16
atr	2180	268	274	1341	297	29.09
atr	2105	236	225	1338	306	25.03
atr	2121	273	216	1353	279	26.59
atr	1918	240	216	1186	276	27.58
mus81	1325	153	117	868	187	23.03
mus81	841	83	74	531	153	20.84
mus81	1218	117	122	791	188	22.05
mus81	1233	121	132	803	177	23.21
mus81	494	41	57	320	76	22.33
atr mus81	1441	158	185	916	182	27.62
atr mus81	1160	170	111	747	132	28.20
atr mus81	1176	158	126	745	147	28.10
atr mus81	883	94	109	557	123	26.50
atr mus81	1073	132	126	691	124	27.95
atr mus81	841	101	94	528	118	26.77
atr mus81	721	92	76	440	113	26.93
atr mus81	1331	147	141	831	212	24.68
atr mus81	759	79	85	489	106	24.64
atr mus81	1243	167	118	785	173	26.42

Supplementary Table S12. *3.9* fluorescent seed count data for the wild type, *atr*, *mus81* and *atr mus81* double mutants in Col/Col inbred lines.

Genotype	Total	Green alone	Red alone	Red+Green	Non-colour	cM
Wild type	1247	113	101	829	204	18.96
Wild type	1458	107	141	985	225	18.77
Wild type	913	77	59	611	166	16.21
Wild type	957	84	60	653	160	16.39
Wild type	1114	97	72	762	183	16.54
Wild type	889	77	67	603	142	17.78
Wild type	635	55	54	417	109	18.96
Wild type	1176	94	95	793	194	17.62
atr	1177	96	60	807	214	14.27
atr	1035	55	72	704	204	13.13
atr	1322	80	93	911	238	14.08
atr	1824	118	114	1256	336	13.65
atr	1458	100	84	1032	242	13.54
atr	1786	114	135	1220	317	15.08
atr	1078	68	70	720	220	13.75
mus81	963	63	78	661	161	15.91
mus81	850	74	68	569	139	18.40
mus81	1155	98	93	759	205	18.19
mus81	1455	107	116	995	237	16.73
mus81	781	63	56	528	134	16.62
atr mus81	1390	90	107	946	247	15.35
atr mus81	902	70	46	610	176	13.81
atr mus81	1411	91	100	942	278	14.60
atr mus81	1258	94	88	842	234	15.70
atr mus81	1337	96	80	906	255	14.17
atr mus81	1162	87	61	809	205	13.67
atr mus81	1441	103	87	1017	234	14.19
atr mus81	1098	69	62	743	224	12.74

Supplementary Table S13. *420* fluorescent seed count data for the wild type, *atr*, *fancd2* and *atr fancd2* double mutants in Col/Col inbred lines.

Genotype	Total	Green alone	Red alone	Red+Green	Non-colour	cM
Wild type	2182	220	216	1380	366	22.52
Wild type	2361	248	220	1570	323	22.31
Wild type	1371	124	130	924	193	20.66
Wild type	790	77	79	523	111	22.21
Wild type	2315	213	234	1485	383	21.65
Wild type	2405	221	231	1555	398	21.00
Wild type	2201	213	198	1450	340	20.85
Wild type	2388	218	217	1595	358	20.27
Wild type	2414	244	211	1588	371	21.07
Wild type	1870	144	204	1236	286	20.77
Wild type	2223	180	207	1499	337	19.26
Wild type	2282	202	220	1481	379	20.62
atr	1961	240	224	1239	258	27.42
atr	1326	145	137	849	195	24.19
atr	1660	222	223	1020	195	31.89
atr	1231	150	143	758	180	27.61
atr	1160	123	127	746	164	24.57
atr	1208	128	144	767	169	25.86
atr	1941	462	430	987	62	28.44
fancd2	1047	101	100	675	171	21.51
fancd2	717	71	64	473	109	21.04
fancd2	975	113	118	606	138	27.46
fancd2	940	86	72	637	145	18.52
fancd2	1588	165	156	1029	238	22.82
fancd2	1361	143	115	877	226	21.20
fancd2	1512	160	144	994	214	22.68
fancd2	1723	171	181	1090	281	23.10
fancd2	1625	172	154	1068	231	22.62
fancd2	1962	208	184	1258	312	22.51
fancd2	2068	226	212	1309	321	24.08
atr fancd2	1110	109	127	712	162	24.19
atr fancd2	2118	222	247	1354	295	25.36
atr fancd2	1923	230	197	1215	281	25.44
atr fancd2	2196	219	278	1386	313	26.02
atr fancd2	1621	195	165	1019	242	25.45
atr fancd2	1592	160	178	1008	246	24.15
atr fancd2	1657	205	189	1020	243	27.58

atr fancd2	1092	145	116	677	154	27.75
atr fancd2	1727	201	160	1099	267	23.72
atr fancd2	2068	225	228	1286	329	25.04

Supplementary Table S14. *3.9* fluorescent seed count data for the wild type, *atr*, *fancd2* and *atr fancd2* double mutants in Col/Col inbred lines.

Genotype	Total	Green alone	Red alone	Red+Green	Non-colour	cM
Wild type	2467	240	169	1631	427	18.24
Wild type	2295	192	152	1553	398	16.32
Wild type	2205	166	178	1502	359	17.06
Wild type	1951	141	147	1347	316	16.05
Wild type	2264	179	206	1484	395	18.77
Wild type	2467	240	169	1631	427	18.24
Wild type	2295	192	152	1553	398	16.32
Wild type	2205	166	178	1502	359	17.06
Wild type	1951	141	147	1347	316	16.05
Wild type	2264	179	206	1484	395	18.77
atr	1913	130	123	1298	362	14.24
atr	2042	113	161	1407	361	14.46
atr	1975	112	117	1362	384	12.36
atr	2105	128	155	1461	361	14.49
atr	2135	132	175	1459	369	15.60
atr	2377	137	157	1581	502	13.25
atr	2178	132	154	1497	395	14.13
atr	2248	164	175	1484	425	16.43
fancd2	1587	95	89	1122	281	12.36
fancd2	1670	105	87	1152	326	12.25
fancd2	1611	95	66	1122	328	10.55
fancd2	1331	77	88	892	274	13.28
fancd2	1570	97	82	1122	269	12.14
fancd2	1473	91	87	1013	282	12.92
atr fancd2	2086	107	140	1425	414	12.64
atr fancd2	1927	125	116	1310	376	13.40
atr fancd2	1703	118	111	1159	315	14.50
atr fancd2	1866	93	90	1276	407	10.34
atr fancd2	1999	113	94	1363	429	10.96
atr fancd2	2211	125	167	1501	418	14.22
atr fancd2	1899	126	136	1269	368	14.91
atr fancd2	1864	88	118	1302	356	11.74
atr fancd2	1944	137	107	1340	360	13.46
atr fancd2	1856	113	108	1279	356	12.72

Supplementary Table S15. *3.9* fluorescent seed count data for the wild type, *atr*, *zyp1* and *atr zyp1* double mutants in Col/Col inbred lines.

Genotype	Total	Green alone	Red alone	Red+Green	Non-colour	cM
Wild type	1889	129	132	1296	332	14.93
Wild type	2075	153	140	1426	356	15.29
Wild type	1793	106	139	1239	309	14.75
Wild type	2184	191	166	1448	379	17.96
Wild type	1753	142	126	1146	339	16.68
Wild type	1742	124	126	1169	323	15.56
Wild type	1855	166	155	1203	331	19.14
Wild type	1778	131	122	1195	330	15.42
atr	2275	141	129	1535	470	12.67
atr	2051	152	123	1432	344	14.45
atr	2068	135	125	1392	416	13.48
atr	1950	124	127	1331	368	13.83
atr	2257	152	137	1535	433	13.75
zyp1	2108	166	143	1431	368	15.93
zyp1	2159	112	97	1527	423	10.20
zyp1	2054	132	132	1375	415	13.81
zyp1	2108	113	107	1471	417	11.05
zyp1	2062	122	90	1415	435	10.87
zyp1	2004	106	107	1385	406	11.26
zyp1	1956	110	112	1345	389	12.08
atr zyp1	2123	87	125	1485	426	10.54
atr zyp1	1840	85	76	1293	386	9.17
atr zyp1	2068	91	92	1460	425	9.28
atr zyp1	1818	86	79	1302	351	9.53
atr zyp1	2157	101	98	1481	477	9.70

Supplementary Table S16. *420* fluorescent seed count data for the wild type in recombinant lines with differing pattern of heterozygosity. The following recombinant backgrounds were used: 'HOM-HOM' that are Col/Col inbred throughout the genome. 'HET-HET' that are Col/Ct heterozygous throughout the genome. 'HET-HOM' where the *420* region is Col/Ct heterozygous and the remainder of chromosome 3 is Col/Col homozygous. and 'HOM-HET' where *420* is Col/Col homozygous and the remainder of chromosome 3 is Col/Ct heterozygous.

Cross	Genotype	Total	Green alone	Red alone	Red+Green	Non-colour	cM
HOM-HOM	Wild type	1997	164	176	1301	356	18.79
HOM-HOM	Wild type	1469	122	108	968	271	17.12
HOM-HOM	Wild type	2031	165	155	1344	367	17.24
HOM-HOM	Wild type	2167	186	153	1448	380	17.11
HOM-HOM	Wild type	2287	224	199	1470	394	20.62
HOM-HOM	Wild type	2226	188	177	1451	410	18.02
HOM-HOM	Wild type	2012	152	151	1332	377	16.41
HOM-HOM	Wild type	1743	150	126	1147	320	17.34
HOM-HOM	Wild type	1802	131	145	1202	324	16.71
HOM-HOM	Wild type	2376	210	211	1526	429	19.65
HOM-HOM	Wild type	2483	221	208	1675	379	19.1
HOM-HOM	Wild type	1620	154	134	1091	241	19.72
HET-HET	Wild type	2056	146	135	1390	385	14.76
HET-HET	Wild type	2127	155	164	1400	408	16.33
HET-HET	Wild type	2116	147	149	1406	414	15.13
HET-HET	Wild type	1847	115	159	1248	325	16.14
HET-HET	Wild type	1850	151	135	1225	339	16.88
HET-HET	Wild type	1674	113	134	1126	301	16.04
HET-HET	Wild type	2096	140	145	1454	357	14.67
HET-HET	Wild type	1954	133	137	1296	388	14.93
HET-HET	Wild type	2140	161	136	1445	398	15.00
HET-HOM	Wild type	2364	264	259	1511	330	25.3
HET-HOM	Wild type	2268	257	295	1381	335	28.4
HET-HOM	Wild type	2418	298	265	1432	423	26.9
HET-HOM	Wild type	2138	271	239	1355	273	27.7
HET-HOM	Wild type	2512	299	259	1495	459	25.5
HET-HOM	Wild type	1914	238	232	1222	222	28.7
HET-HOM	Wild type	2183	232	266	1402	283	26.3
HET-HOM	Wild type	2148	236	239	1378	295	25.3
HET-HOM	Wild type	2122	236	287	1332	267	28.8
HOM-HET	Wild type	2024	138	140	1344	402	14.83

HOM-HET	Wild type	2361	94	147	1666	454	10.79
HOM-HET	Wild type	2061	87	118	1438	418	10.5
HOM-HET	Wild type	1551	67	86	1089	309	10.41
HOM-HET	Wild type	2258	98	122	1569	469	10.27
HOM-HET	Wild type	1898	133	108	1303	354	13.63

Supplementary Table S17. *420* fluorescent seed count data for the *atr* single mutant in Col/Ct recombinant lines with differing patterns of heterozygosity.

Cross	Genotype	Total	Green alone	Red alone	Red+Green	Non-colour	cM
HOM-HOM	*atr*	2319	287	264	1461	307	27.56
HOM-HOM	*atr*	2389	289	261	1518	321	26.55
HOM-HOM	*atr*	1128	114	109	719	186	22.24
HOM-HOM	*atr*	2362	269	253	1498	342	25.30
HOM-HOM	*atr*	2324	310	251	1432	331	28.08
HOM-HOM	*atr*	2484	280	266	1599	339	25.14
HOM-HOM	*atr*	2218	277	238	1408	295	26.81
HET-HET	*atr*	2490	239	220	1614	417	20.54
HET-HET	*atr*	2475	229	225	1623	398	20.43
HET-HET	*atr*	2476	271	245	1562	398	23.63
HET-HET	*atr*	2471	264	246	1567	394	23.37
HET-HET	*atr*	2509	266	233	1602	408	22.40
HET-HET	*atr*	2322	224	192	1498	408	19.89
HET-HET	*atr*	2505	229	212	1684	380	19.51
HET-HET	*atr*	2496	244	231	1594	427	21.30
HET-HET	*atr*	2484	245	225	1591	423	21.16
HET-HET	*atr*	2417	223	220	1593	381	20.41
HET-HET	*atr*	2301	186	238	1506	371	20.54
HET-HET	*atr*	2301	215	206	1530	350	20.37
HET-HET	*atr*	2262	206	211	1477	368	20.55
HET-HET	*atr*	2490	239	220	1614	417	20.54
HET-HOM	*atr*	2388	300	317	1446	325	30.48
HET-HOM	*atr*	1552	195	190	976	191	29.02
HET-HOM	*atr*	2270	294	273	1425	278	29.26
HET-HOM	*atr*	2352	306	312	1427	307	31.12
HET-HOM	*atr*	2395	341	281	1469	304	30.68
HET-HOM	*atr*	2322	282	329	1430	281	31.17
HET-HOM	*atr*	2164	280	264	1333	287	29.49
HET-HOM	*atr*	2403	343	266	1487	307	29.78
HET-HOM	*atr*	1198	146	149	756	147	28.76
HET-HOM	*atr*	1630	198	224	1013	195	30.56
HET-HOM	*atr*	2161	264	260	1321	316	28.23
HET-HOM	*atr*	2178	301	262	1355	260	30.50
HOM-HET	*atr*	2281	119	180	1548	434	14.10
HOM-HET	*atr*	2323	162	145	1576	440	14.23
HOM-HET	*atr*	2275	108	150	1552	465	12.07

HOM-HET	*atr*	2477	193	169	1682	433	15.87
HOM-HET	*atr*	2439	139	166	1649	485	13.40
HOM-HET	*atr*	2266	134	193	1540	399	15.66
HOM-HET	*atr*	2378	157	128	1643	450	12.80
HOM-HET	*atr*	2343	151	155	1565	472	14.05

Supplementary Table S18. *420* fluorescent seed count data for the *fancm zip4* double mutant in Col/Ct recombinant lines with differing patterns of heterozygosity.

Cross	Genotype	Total	Green alone	Red alone	Red+ Green	Non-colour	cM
HOM-HOM	*fancm zip4*	2163	320	277	1340	226	33.07
HOM-HOM	*fancm zip4*	2192	339	320	1318	215	36.86
HOM-HOM	*fancm zip4*	2226	321	358	1315	232	37.56
HOM-HOM	*fancm zip4*	747	101	105	459	82	33.03
HOM-HOM	*fancm zip4*	1730	249	241	1060	180	34.16
HOM-HOM	*fancm zip4*	1896	286	278	1126	206	36.36
HOM-HOM	*fancm zip4*	1864	257	278	1136	193	34.73
HET-HET	*fancm zip4*	2044	159	147	1352	386	16.30
HET-HET	*fancm zip4*	2068	155	156	1370	387	16.38
HET-HET	*fancm zip4*	1918	143	115	1294	366	14.50
HET-HET	*fancm zip4*	1518	113	117	1040	248	16.52
HET-HET	*fancm zip4*	1560	139	124	1022	275	18.59
HET-HET	*fancm zip4*	1724	131	185	1138	270	20.41
HET-HET	*fancm zip4*	1892	116	157	1264	355	15.65
HET-HET	*fancm zip4*	1033	95	73	691	174	17.86
HET-HOM	*fancm zip4*	2268	126	116	1572	454	11.31
HET-HOM	*fancm zip4*	2145	124	116	1457	448	11.90
HET-HOM	*fancm zip4*	2410	148	166	1679	417	14.01
HET-HOM	*fancm zip4*	2102	118	147	1420	417	13.52
HET-HOM	*fancm zip4*	2308	149	146	1608	405	13.72
HET-HOM	*fancm zip4*	2270	132	129	1549	460	12.25
HET-HOM	*fancm zip4*	2283	147	178	1545	413	15.43
HET-HOM	*fancm zip4*	2342	180	149	1561	452	15.20
HET-HOM	*fancm zip4*	2187	123	155	1485	424	13.64
HET-HOM	*fancm zip4*	2248	151	122	1543	432	12.99
HET-HOM	*fancm zip4*	1996	102	124	1367	403	12.05
HOM-HET	*fancm zip4*	1477	233	257	847	140	41.99
HOM-HET	*fancm zip4*	1785	261	274	1096	154	36.71
HOM-HET	*fancm zip4*	1698	289	278	978	153	42.37
HOM-HET	*fancm zip4*	1955	277	331	1164	183	38.52
HOM-HET	*fancm zip4*	1850	281	297	1124	148	38.75
HOM-HET	*fancm zip4*	2037	318	344	1196	179	40.84
HOM-HET	*fancm zip4*	1929	323	312	1108	186	41.55
HOM-HET	*fancm zip4*	1999	324	325	1159	191	40.78
HOM-HET	*fancm zip4*	1283	222	225	730	106	44.94
HOM-HET	*fancm zip4*	2225	362	377	1276	210	42.06

HOM-HET	*fancm zip4*	1885	291	321	1107	166	40.78
HOM-HET	*fancm zip4*	2123	308	330	1295	190	36.84
HOM-HET	*fancm zip4*	503	86	79	297	41	41.35
HOM-HET	*fancm zip4*	1369	221	245	783	120	43.50

Supplementary Table S19. *420* fluorescent seed count data for the *atr fancm zip4* triple mutant in Col/Ct recombinant lines with differing patterns of heterozygosity.

Cross	Genotype	Total	Green alone	Red alone	Red+ Green	Non-colour	cM
HOM-HOM	*atr fancm zip4*	2108	310	339	1265	194	38.01
HOM-HOM	*atr fancm zip4*	2293	375	376	1342	200	41.27
HOM-HOM	*atr fancm zip4*	2208	382	377	1280	169	44.10
HOM-HOM	*atr fancm zip4*	1562	229	265	919	149	39.38
HOM-HOM	*atr fancm zip4*	1746	264	290	1044	148	39.55
HOM-HOM	*atr fancm zip4*	2271	361	394	1334	182	42.11
HOM-HOM	*atr fancm zip4*	2158	353	361	1231	213	41.84
HOM-HOM	*atr fancm zip4*	2422	406	396	1387	233	41.88
HOM-HOM	*atr fancm zip4*	2398	376	420	1383	219	42.02
HOM-HOM	*atr fancm zip4*	2259	337	356	1377	189	37.83
HET-HET	*atr fancm zip4*	1978	174	168	1303	333	19.12
HET-HET	*atr fancm zip4*	1847	194	174	1184	295	22.44
HET-HET	*atr fancm zip4*	1898	170	171	1246	311	19.96
HET-HET	*atr fancm zip4*	2250	205	180	1467	398	18.90
HET-HET	*atr fancm zip4*	2315	197	201	1543	374	19.00
HET-HET	*atr fancm zip4*	1760	182	155	1153	270	21.45
HET-HET	*atr fancm zip4*	2111	191	169	1424	327	18.83
HET-HET	*atr fancm zip4*	2027	171	234	1313	309	22.51
HET-HET	*atr fancm zip4*	1504	132	147	987	238	20.69
HET-HET	*atr fancm zip4*	2117	215	202	1359	341	22.15
HET-HOM	*atr fancm zip4*	2412	188	175	1586	463	16.39
HET-HOM	*atr fancm zip4*	2296	172	188	1510	426	17.15
HET-HOM	*atr fancm zip4*	1829	113	133	1241	342	14.50
HET-HOM	*atr fancm zip4*	2232	174	178	1474	406	17.26
HET-HOM	*atr fancm zip4*	2033	128	157	1408	340	15.17
HET-HOM	*atr fancm zip4*	2569	208	179	1741	441	16.41
HET-HOM	*atr fancm zip4*	2025	127	158	1363	377	15.23
HET-HOM	*atr fancm zip4*	2225	146	165	1505	409	15.12
HET-HOM	*atr fancm zip4*	2319	147	175	1547	450	15.01
HET-HOM	*atr fancm zip4*	2417	164	188	1606	459	15.81
HOM-HET	*atr fancm zip4*	2126	330	396	1235	165	43.69
HOM-HET	*atr fancm zip4*	2303	396	400	1288	219	44.44
HOM-HET	*atr fancm zip4*	2162	342	376	1277	167	42.05
HOM-HET	*atr fancm zip4*	2283	367	407	1344	165	43.26
HOM-HET	*atr fancm zip4*	2037	341	336	1203	157	42.10
HOM-HET	*atr fancm zip4*	2256	370	362	1329	195	40.75

HOM-HET	*atr fancm zip4*	2146	367	372	1244	163	44.21
HOM-HET	*atr fancm zip4*	2167	393	358	1242	174	44.60
HOM-HET	*atr fancm zip4*	2175	345	381	1288	161	42.34
HOM-HET	*atr fancm zip4*	1836	328	320	1042	146	45.77

Supplementary Table S20. Primers used for mutant genotyping.

Primer name	Gene ID	Primer sequence	Comments
sni1-dCAPS-F	AT4G18470	TGGTTTTGTTTTGCAGGCTTGGTCACCAT	dCAPS – digestion with Tsp509I
sni1-dCAPS-R		GTGAAATCTAGCTTAAGAACATGAGCAAGA	
mus81-LP	AT4G30870	AGTGAATCTGATAGTGAGTG	
mus81-RP		GCAGCATCAATAAGCTCTTG	
mus81-BP(GK)		GACCATCATACTCATTGCTG	
zip4-LP	AT1G56590	TTGCTACCTTGGGCTCTCTC	
zip4-RP		ATTCTGTTCTCGCTTTCCAG	
fancm-dCAPS-F	AT1G35530	ACAATATATGTTTCGTGCAGGTAAGACATTGGAAG	dCAPS – digestion with MboII
fancm-dCAPS-R		CACCAATAGATGTTGCGACAAT	
atr2LP	AT5G40820	GAGCAAATGCAAGAACTCTGG	
atr2RP		ACTTCAAGGGTTCCGATGTTC	
fancd2LP	AT4G14970	CATGTACATGAATGGGGATCC	
fancd2RP		TGAAAACCTCATAGACCTGCG	
zyp1-288A	AT1G22260/ AT1G22275	ATAGATCGATTTCGTCATCT	dCAPS – digestion with MboI
zyp1-288B		GAGGTGAAATATGAATCTGCT	
LBb1.3		ATTTTGCCGATTTCGGAAC	Genotyping of SALK insertional mutants
BAR-F		GGCAGCTGGACTTCAGCCT	genotyping of CRISPR/Cas9 construct
pFGC-2R		AGCCACGCACATTTAGGATA	

Supplementary Table S21. Primers used for ATR targeting in Ct and L*er* (gRNA) and *atr* CRISPR/Cas9 mutant Sanger sequencing and genotyping.

**Primer name**	**Primer sequence**
U6-ATR-gRNA	TAATCTATTGAATACCTACGCAATCACTACTTCGACTCTAGCTGT
U3-ATR-gRNA	CTCCGGCTAACAACCTCCGTGACCCTTGATGCTTTCTATGCA
ATR-F	GCGAGAGAGCTTCTGCGATT
ATR-R	GCTAAGAATCATCCAAGGGAACAG

Supplementary Table S22. Primers used for genome sequencing and *seed-typing* library preparation.

Name	Oligonucleotide sequence
Tn5ME-A	TCGTCGGCAGCGTCAGATGTGTATAAGAGACAG
Tn5ME-B	GTCTCGTGGGCTCGGAGATGTGTATAAGAGACAG
Tn5MErev	[phos]CTGTCTCTTATACACATCT
N701	CAAGCAGAAGACGGCATACGAGATTCGCCTTAGTCTCGTGGGCTCGG
N702	CAAGCAGAAGACGGCATACGAGATCTAGTACGGTCTCGTGGGCTCGG
N703	CAAGCAGAAGACGGCATACGAGATTTCTGCCTGTCTCGTGGGCTCGG
N704	CAAGCAGAAGACGGCATACGAGATGCTCAGGAGTCTCGTGGGCTCGG
N705	CAAGCAGAAGACGGCATACGAGATAGGAGTCCGTCTCGTGGGCTCGG
N706	CAAGCAGAAGACGGCATACGAGATCATGCCTAGTCTCGTGGGCTCGG
N707	CAAGCAGAAGACGGCATACGAGATGTAGAGAGGTCTCGTGGGCTCGG
N708	CAAGCAGAAGACGGCATACGAGATCCTCTCTGGTCTCGTGGGCTCGG
N709	CAAGCAGAAGACGGCATACGAGATAGCGTAGCGTCTCGTGGGCTCGG
N710	CAAGCAGAAGACGGCATACGAGATCAGCCTCGGTCTCGTGGGCTCGG
N711	CAAGCAGAAGACGGCATACGAGATTGCCTCTTGTCTCGTGGGCTCGG
N712	CAAGCAGAAGACGGCATACGAGATTCCTCTACGTCTCGTGGGCTCGG
N714	CAAGCAGAAGACGGCATACGAGATTCATGAGCGTCTCGTGGGCTCGG
N715	CAAGCAGAAGACGGCATACGAGATCCTGAGATGTCTCGTGGGCTCGG
N716	CAAGCAGAAGACGGCATACGAGATTAGCGAGTGTCTCGTGGGCTCGG
N718	CAAGCAGAAGACGGCATACGAGATGTAGCTCCGTCTCGTGGGCTCGG
N719	CAAGCAGAAGACGGCATACGAGATTACTACGCGTCTCGTGGGCTCGG
N720	CAAGCAGAAGACGGCATACGAGATAGGCTCCGGTCTCGTGGGCTCGG
N721	CAAGCAGAAGACGGCATACGAGATGCAGCGTAGTCTCGTGGGCTCGG
N722	CAAGCAGAAGACGGCATACGAGATCTGCGCATGTCTCGTGGGCTCGG
N723	CAAGCAGAAGACGGCATACGAGATGAGCGCTAGTCTCGTGGGCTCGG
N724	CAAGCAGAAGACGGCATACGAGATCGCTCAGTGTCTCGTGGGCTCGG
N726	CAAGCAGAAGACGGCATACGAGATGTCTTAGGGTCTCGTGGGCTCGG
N727	CAAGCAGAAGACGGCATACGAGATACTGATCGGTCTCGTGGGCTCGG
S501	AATGATACGGCGACCACCGAGATCTACACTAGATCGCTCGTCGGCAGCGTC
S502	AATGATACGGCGACCACCGAGATCTACACCTCTCTATTCGTCGGCAGCGTC
S503	AATGATACGGCGACCACCGAGATCTACACTATCCTCTTCGTCGGCAGCGTC
S504	AATGATACGGCGACCACCGAGATCTACACAGAGTAGATCGTCGGCAGCGTC
S505	AATGATACGGCGACCACCGAGATCTACACGTAAGGAGTCGTCGGCAGCGTC
S506	AATGATACGGCGACCACCGAGATCTACACACTGCATATCGTCGGCAGCGTC

S507	AATGATACGGCGACCACCGAGATCTACACAAGGAGTATCGTCGGCAGCGTC
S508	AATGATACGGCGACCACCGAGATCTACACCTAAGCCT TCGTCGGCAGCGTC
S509	AATGATACGGCGACCACCGAGATCTACACGGCTACTCTCGTCGGCAGCGTC
S510	AATGATACGGCGACCACCGAGATCTACACCCTCAGACTCGTCGGCAGCGTC
S511	AATGATACGGCGACCACCGAGATCTACACTCCTTACGTCGTCGGCAGCGTC
S512	AATGATACGGCGACCACCGAGATCTACACACGCGTGGTCGTCGGCAGCGTC
S513	AATGATACGGCGACCACCGAGATCTACACGGAACTCCTCGTCGGCAGCGTC
S514	AATGATACGGCGACCACCGAGATCTACACTGGCCATGTCGTCGGCAGCGTC
S515	AATGATACGGCGACCACCGAGATCTACACGAGAGATTTCGTCGGCAGCGTC
S516	AATGATACGGCGACCACCGAGATCTACACCGCGGTTATCGTCGGCAGCGTC
S517B	AATGATACGGCGACCACCGAGATCTACACGACCGCCATCGTCGGCAGCGTC
S518	AATGATACGGCGACCACCGAGATCTACACTAAGATGGTCGTCGGCAGCGTC
S519	AATGATACGGCGACCACCGAGATCTACACATTGACATTCGTCGGCAGCGTC
S520	AATGATACGGCGACCACCGAGATCTACACAGCCAACTTCGTCGGCAGCGTC
S521	AATGATACGGCGACCACCGAGATCTACACTACTAGGTTCGTCGGCAGCGTC
S522	AATGATACGGCGACCACCGAGATCTACACTCACGGTTTCGTCGGCAGCGTC
S523	AATGATACGGCGACCACCGAGATCTACACTGTAATGATCGTCGGCAGCGTC
S524	AATGATACGGCGACCACCGAGATCTACACCACGTCAGTCGTCGGCAGCGTC

Supplementary Table S23. Primers used for LR-PCR in *seed-typing*.

**Primer name**	**Primer sequence**	**Comments**
ChP_1_Col, Forward	TGATAGTTCTCCGAAGAATACTTCCAT	LR-PCR of *ChP* Region 1, 8787 bp in Col
ChP_1_Col, Reverse	ACGACCTTCTTATTTGCCAATTCAT	
ChP_2_Col, Forward	GATTGGTTTAGCTGGTTGGATCCG	LR-PCR of ChP Region 2,10235 bp in Col
ChP_2_Col, Reverse	TACTTTTGCTCCTCACACCCAAGA	
ChP_3_Col, Forward	AGCTTCCTCTGCCACTAAATCACA	LR-PCR of ChP Region 3,10168 bp in Col
ChP_3_Col, Reverse	TTTTCAGACAAACTCCAATTTCACAG	
