# Supplementary material for: The kinase ATR controls meiotic crossover distribution at the genome scale in Arabidopsis
Source: Plant Cell. 2024 Oct 29;37(1):koae292. doi: 10.1093/plcell/koae292 (PMC11663591; doi:10.1093/plcell/koae292)
Supplement: koae292_Supplementary_Data [file koae292_supplementary_data.zip › tpc.11132.2024-s07.pdf]

THE PLANT CELL

ATR controls meiotic crossover distribution at the genome scale in Arabidopsis

Longfei Zhu, Julia Dluzewska, Nadia Fernández-Jiménez, Rajeev Ranjan, Alexandre Pelé, Wojciech Dziegielewski, Maja Szymanska-Lejman, Karolina Hus, Julia Górna, Monica Pradillo, and Piotr Ziolkowski

Review Timeline:

Submission Date:	26-Jan-2024
Editorial Decision:	27-Feb-2024
Resubmission Received:	16-Aug-2024
Editorial Decision:	09-Sep-2024
Revision Received:	07-Oct-2024
Accepted:	14-Oct-2024

Prof. Piotr A. Ziolkowski
Uniwersytet im Adama Mickiewicza w Poznaniu
Poznan 61-614
Poland

Dear Piotr:

Thank you for choosing to send your manuscript entitled "ATR controls meiotic crossover distribution at the local and global scales in Arabidopsis" for consideration at The Plant Cell. Your submission has been evaluated by members of the editorial board as well as expert reviewers in your field, and we regret to inform you that we are not able to recommend the publication of this manuscript in its current form. We have had input from multiple scientists and we have solicited post-review comments.

During the post-review consultation session, we also agreed that if you could address the major points raised by the reviewers by new experiments, we would welcome a resubmission. This may be treated as a new submission, but we would attempt to use at least some of the same reviewers. Nevertheless, reviewers will be asked to assess as a new manuscript (i.e. are the claims fully supported by the data and do the results presented move the field forward?), and not only whether previous reviewer comments have been addressed.

The most crucial feedback guiding our decision highlighted two main issues with your manuscript: firstly, the absence of appropriate controls for several experiments (including whole genome recombination, hot spot analyses, and gH2AX IF); secondly, the need to further analyze the atr recombination landscape (such as comparing it with already published data on other genetic contexts demonstrating similar CO distribution changes and clarifying the hot spot usage analysis).

It will be important to convince the editors and reviewers that the study adds significant new understanding(s) of mechanisms or processes, or otherwise represents a significant advance in the field, and that the major claims made are fully justified by the data presented. This includes careful consideration and explanation of the various controls used in experiments, the extent and manner of replication, and the statistical analyses used. Sampling methods and the nature of "biological replicates" should be described precisely (i.e. different plants, parts of plants, pooled tissue, independent pools of tissue, sampled at different times, etc.), along with a clear description of and rationale for any statistical analyses conducted. The reader should know exactly what was sampled; what forms the basis of the calculation of any means and other statistical variables and parameters reported. This is also necessary to ensure that proper statistical analysis was conducted.

The Plant Cell now requires authors to complete and submit an author revisions checklist upon submission of a revised manuscript. The aim of the checklist is to aid authors in preparing a high-quality manuscript, facilitate the review and assessment of revised manuscripts, and help to ensure that journal standards are maintained across the board. If your manuscript is accepted, the completed checklist will be published as supplemental material attached to the article online. Please download a copy of the checklist (a fillable PDF form) at this link, for submission with your revised manuscript: https://tpc.msubmit.net/html/Author_Revisions_Checklist.pdf.

Note also that supplemental materials should be restricted to large datasets and tables, presentation of replicates, and validation of reagents, methods, or genotypes. Any data that are used to support the main claims must be in the main manuscript. Supplemental figure legends must indicate what figure in the main manuscript is supported by the supplemental data presented.

We thank you for your interest in and support of The Plant Cell. We hope we will be able to render a more positive decision on future work.

Sincerely,

The Plant Cell Editorial Board

--- Reviewer comments:

Reviewer #1 (Comments for the Author):

Longfei Zhu and authors present their results on the characterization of the meiosis behavior of the atr mutant in Arabidopsis. In a previous paper (Zhu et al., 2021), they had identified an increase in the recombination frequency at the '420' locus in the sni1 atr double mutant compared to the single sni1 mutant, and in this paper, they characterize further the effect of atr on the

recombination landscape. The paper is well written and the figures are nicely laid out.

While no difference in the number of DSBs is observed (based on gH2AX staining), more crossovers are observed using fluorescent markers segregation-based measures in intra-arm intervals, while less crossovers are observed in intervals closers to the centromeres. This delocalization of crossover events seems to affect both class I and class II crossovers, as the two pathways are both required for and affect by this effect. This suggests that ATR regulates recombination steps upstream of class I/class II actors, probably at the invasion step.

My major comment is in regard to the use of appropriate controls for both the whole genome and the hotpost crossover detection by sequencing experiments. In both cases, the authors generated the atr data de novo for the paper, but did not generate any new wild-type data (sisters of the atr mutant), and instead used previously published data (Rowan et al, 2019 for the genome wide data and their own published data for the hotspots Szymanska-Lejman et al., 2023). This is concerning, and I invite the authors to justify this choice thoroughly. Although I understand that whole-genome sequencing experiments are expensive, I would strongly advise the authors to consider sequencing the F2 descendants of the wild-type sister plants of their atr mutant grown in the same controlled environment, and include these new data in the paper as a proper control. I have to request at the very least sequencing of wild-type sister recombinant ESILs lines for the data included in Figure 3E (blue wild-type data). All meiotic mutant lines are best kept as heterozygous seed stocks, so the authors should have access to the sister wild-type plants. I understand that it requires crosses that might not have been performed at the time, but I really strongly encourage the authors to go back and generate the appropriate material for proper comparison.

Points of further discussion:
- How would you explain that we do not see a difference in recombination frequency at the ChP and BT hotspots in a pure Col background (Fig 3B)? You discuss the difference between Col/Col and Col/Ler in the context of the MLH1 focus count in your discussion, but I don't recall any discussion of this figure.
- Similarly, why do we see a difference between WT and atr in Col/Ler context at the ChP hotspot but not at the BT one?

Minor comments:
Figure 1
- Any statistical correction for multiple testing? I doubt that it will affect the conclusions at all, but it is good practice. You can additionally remove the exact p-value numbers (which are not very meaningful anyway) and replace them with **/*** when reaching appropriate thresholds
- I would appreciate a simple scheme to indicate the location on chromosomes 1 and 3 of all measured intervals (with appropriate scale, see below Figure 3A).
- '420' is jargon for anyone but us! Please use appropriate wording in figure legends.

Figure 2
- Legend: what is the window size for binning crossover events? (SNPs is 300kb)

Figure 3
- Fig 3A: Please add a scale (mm to Mb) or exact positions (in Mb, if the scheme is up to scale) of CEN, BT, ChP and pericentromeric boxes.
- What definition of "pericentromeric" did the authors choose? Based on what kind of data?
- Fig 3E: could you move the names of the hotspots (cute dogs as they are) below the position in Mb of the x axis? It looks very crowded over imposed on the graph itself.
- Fig 3F: I would advise using stacked bar graph, as you are comparing proportions of events in all three categories.
- Fig 3D is barely mentioned in the text, it could be moved to supplementary
- Fig 3G: same comment for multiple testing and p-values as in Fig 1.

Figure 4
- Fig 4A: I am not sure I understand this figure. Are you comparing the 52k crossover events in the wild type to your much smaller data set in atr? Are you normalizing for the total number of crossovers? I would guess not because the atr mutant shows a bit more crossovers than the wild type based on Fig 2B. I think this "pseudohotspot" is an interesting approach, but I am not sure this statistics and this representation is the most suitable to convey your point.

Figure 5
- Legend reads "crossover interference is weaker" although all comparisons are non-significant and the text actually says page 13 line 337 that "interference is not affected in atr"
- There is no figure 5F (anymore?), although the legend still mentions it. I think you can just remove the legend part, the interference data from FTL on chromosome 3 are not adding anything more I guess (no difference, or maybe a slight one?)

Figure 6
- Maybe you could consider merging Figure 5 and 6 to convey the point that both pathways are similarly affected by the atr effect? You could move panels 5E and 6C to supplementary. This is a proposition and a personal preference; the authors are

free to not consider it.

Figure 7
- Legend: title should read "remodeling of crossover distribution" (the distribution is remodeled, not the crossovers)
- P-values: see above

Figure 8
- P-values: see above

Figure 9
- For which comparisons are the p-values? To wild type? To avoid over-crowding of the diagram, you could mention in the legend that "all comparisons are statistically significant (compared to xx) except when noted"

Main text
- Page 3 line 69-74: I am of the opinion that not all second-end captures give rise to nice and simple dHj (see de Muyt et al., 2012 for instance). Moreover, we have no way of knowing whether "a majority" of D-loops are dissolved through SDSA. What we do know is that there are more complex events that we first considered (see Ahuja et al., 2021), so a lot might be happening at the invasion step. Most D-loop are converted to non-crossovers (or inter-sister events), that much we know. Please reword this bit.
- In the last paragraph of the introduction, you already mention pseudo-hotspots. I was not familiar with this approach before reading your manuscript, and it felt weird reading about it at this time in the text. I would say less at this point (trying to word it without needing to use the pseudo-hotspots?) and keep the full description for the results section.
- If you choose to merge figure 5 and 6, you could merge the respective paragraphs as well.
- I do not like the GBS acronym. It is not a well-known acronym, and I would refrain from using it extensively (use "sequencing data" maybe?). This is a personal preference.
- Please italicize Arabidopsis everywhere, it is a species name.
- Careful about atr not being properly italicized either sometimes (e.g. line 275).
- Line 385: the gene msh4 should be in small caps

I liked this paper and I hope the authors will have the resources to provide us with appropriate controls that will allow their conclusions to be supported accordingly.

Best of luck for the revisions.

Reviewer #2 (Comments for the Author):

In this manuscript, Zhu et al explore the role of the ATR kinase in meiotic crossover formation in Arabidopsis. They conclude that ATR inactivation leads to the redistribution of crossover along chromosomes, with a decrease in centromere-proximal regions and an increase in distal regions. Some interesting results are presented, however, the main conclusions are insufficiently supported as detailed below:
Major comments:

1) In all genome-wide analyses, the atr Col/Ler hybrid is compared with a col/ler wild type previously described and produced in a different lab, without internal wild type controls. This is problematic as environmental factors or potential differences in the genetic background could affect crossover number and distribution. The author must include an analysis of the Col/Ler wild type from the same lineage and in their lab conditions.
2) The authors use immunodetection of γH2A.X to quantify DSB formation. However, the specificity of the staining/antibody is not supported. A negative control is essential. One possibility is to use a known DSB defective mutant (e.g. spo11) and show that, when done in parallel, foci are observed in wild types and not in mutants.
3) Line 238-250. While it is clear that all three hotspots have reduced activity I'm not convinced that there is any significant difference in the contribution of the different hotspots. A proper test should be performed (e.g. Chi-square) to test if the proportion of CO in the three hotspots is different between wild-type and mutant. With n=68, my prediction is that this will not be significantly different. All parts of the manuscript using this conclusion (differences in hotspot usage) should be revised.
4) L251-277. I'm not convinced of the pertinence of this analysis. First, this is not an analysis of hot spots (as recognized by the usage of the word „pseudo-hotspot" in the text). So the conclusion of „biased hotspot usage" is not supported. At best the analysis indicates how clustered are the COs. Second, the observed effects are quite subtle. For example, 11.7% vs 12.8% of crossover found in the „extremely strong category) is not a large effect. Figure 3A should show the % rather than numbers (and Figure 4B could be omitted). Third, and probably more importantly, I'm afraid that the method could be biased by the fact that the will-type data is a very large part of the „reference data" that defines the „hotspots". Thus relatively cold intervals could have by chance one rare crossover in wild-type, and the interval would be classified „sporadic". Then, the same CO would be counted as

falling in a „sporadic" interval. This effect could inflate the proportion of „sporadic" CO in the wild type, and then largely explain the observations. Finally, and as mentioned in the first point the mutant and wild-type data were produced in different conditions.

5) L289. How many cells were analyzed?

Minor comments
It would be useful to comment on fertility in atr. Reduction of fertility does not necessarily mean a meiotic defect, but full fertility (as shown later in the manuscript) means that meiosis is fine.

In the introduction, the authors navigate between the conserved features of meiosis recombination and focus on arabidopsis. This is fine, but it should be always clear what we are talking about. For example, lines 94-97 this statement and numbers are for Arabidopsis, but this is unclear and should be clearly stated. Please review the entire introduction with this in mind.
Line 180. There is a discrepancy in the number of analysed plants between the figure (n=180) and the text (n=220)
223-224. Please clarify: how amplified? how sequenced?
Figure 2E. What is shown in blue/green/red on the top of the graph?
-
Please provide the catalog number of the γH2A.X antibody
L328. The distribution of class II CO is not random. Independent would be more appropriate.
L343. Is there any reliable Class II crossover markers in non-plant eukaryotes?
L352. Neither an increase nor reduction in class II COs.

Reviewer #3 (Comments for the Author):

This study performs the genetic analysis to better understand the function of Ataxia telangiectasia-mutated and Rad3-related (ATR) kinase in meiotic recombination in Arabidopsis thaliana. ATR kinase in Arabidopsis had been shown to regulate the deposition of DMC1 at meiotic resected breaks but did not appear to affect meiosis overall in a drastic manner unless combined with ATM kinase. The manuscript submitted by Zhu et al. presents a detailed description of the role of ATR kinase in controlling the distribution of meiotic crossovers. The conclusions of the paper are as follows. In the atr mutant, the CO frequency is increased and there is a redistribution with a CO increase in the subtelomeric region and a decrease in the pericentromeric region, the hotspot usage is modified. However, the authors do not find any correlation or causative link between this change in CO distribution and heterozygosity along the chromosome, number of meiotic DSBs, interference measured with MLH1 foci representing class I CO or with components of class I or class II pathways. Overall, this study concludes that the loss of ATR is manifested in the redistribution of CO and hotspot usage, which are attributed to the likely early role of ATR in meiotic recombination. There is a good quantity of data in this paper and the experiments are carried out using well-established methods combining genetics, genomics, and cytology approaches with proper design.

Major concerns:
However, the study remains descriptive without any molecular mechanism identifying how ATR might be controlling the distribution of CO.
1. The claim that ATR controls the number of COs is not evident. Indeed, genome-wide increase of CO is not very evident except in chromosome 3 and to a small extent in chromosome 5, other chromosomes don't show an increase in CO frequency. It is also not very clear if this redistribution of CO is very specific to ATR or if it is a general phenomenon of mutants affecting CO formation. A similar redistribution is observed in asy1+/- heterozygous (Lambing et al 2020 PNAS) and figl1 mutants (Fernandes et al 2018 PNAS).
2. Furthermore, the interference analysis based on MLH1 foci count done at the pachytene stage may not be the best stage when looking for redistribution and may not provide a true representation of chiasma. The dynamics of MLH1 foci has been shown to be modified between Pachytene and Diplotene or Diakinesis and It would be better to look at these later stages (Lambing et al 2020 PNAS, and Li et al 2021 PNAS (HEI10 foci)), where one can measure true redistribution per bivalent and a putative mutant effect on the dynamics of the MLH1 foci.
3. The proposed model for ATR controlling the hotspot usage remains rather correlative than causative. The study should have least included an analysis of the localization of the hotspot regarding their strength. Also, it would have been interesting to analyze the hotspot usage in asy1+/- and figl1 mutant with a similar approach.
The results provided here are likely to be interesting for the meiotic recombination community, but conclusions made possible by the data are of insufficient impact to justify publication in the Plant Cell as compared to a more specialized journal.

Date: 27-Feb-2024 08:46:02

Last Sent: 27-Feb-2024 08:46:02

Triggered By: Redacted

CC: Redacted

BCC: Redacted

Subject: TPC2024-RA-00079 - Decision Letter

Message: Prof. Piotr A. Ziolkowski

Uniwersytet im Adama Mickiewicza w Poznaniu

Poznan 61-614

Poland

Dear Piotr:

Thank you for choosing to send your manuscript entitled "ATR controls meiotic crossover distribution at the local and global scales in Arabidopsis" for consideration at The Plant Cell. Your submission has been evaluated by members of the editorial board as well as expert reviewers in your field, and we regret to inform you that we are not able to recommend the publication of this manuscript in its current form. We have had input from multiple scientists and we have solicited post-review comments.

During the post-review consultation session, we also agreed that if you could address the major points raised by the reviewers by new experiments, we would welcome a resubmission. This may be treated as a new submission, but we would attempt to use at least some of the same reviewers. Nevertheless, reviewers will be asked to assess as a new manuscript (i.e. are the claims fully supported by the data and do the results presented move the field forward?), and not only whether previous reviewer comments have been addressed.

The most crucial feedback guiding our decision highlighted two main issues with your manuscript: firstly, the absence of appropriate controls for several experiments (including whole genome recombination, hot spot analyses, and gH2AX IF); secondly, the need to further analyze the atr recombination landscape (such as comparing it with already published data on other genetic contexts demonstrating similar CO distribution changes and clarifying the hot spot usage analysis).

It will be important to convince the editors and reviewers that the study adds significant new understanding(s) of mechanisms or processes, or otherwise

represents a significant advance in the field, and that the major claims made are fully justified by the data presented. This includes careful consideration and explanation of the various controls used in experiments, the extent and manner of replication, and the statistical analyses used. Sampling methods and the nature of "biological replicates" should be described precisely (i.e. different plants, parts of plants, pooled tissue, independent pools of tissue, sampled at different times, etc.), along with a clear description of and rationale for any statistical analyses conducted. The reader should know exactly what was sampled; what forms the basis of the calculation of any means and other statistical variables and parameters reported. This is also necessary to ensure that proper statistical analysis was conducted.

The Plant Cell now requires authors to complete and submit an author revisions checklist upon submission of a revised manuscript. The aim of the checklist is to aid authors in preparing a high-quality manuscript, facilitate the review and assessment of revised manuscripts, and help to ensure that journal standards are maintained across the board. If your manuscript is accepted, the completed checklist will be published as supplemental material attached to the article online. Please download a copy of the checklist (a fillable PDF form) at this link, for submission with your revised manuscript: https://tpc.msubmit.net/html/Author_Revisions_Checklist.pdf.

Note also that supplemental materials should be restricted to large datasets and tables, presentation of replicates, and validation of reagents, methods, or genotypes. Any data that are used to support the main claims must be in the main manuscript. Supplemental figure legends must indicate what figure in the main manuscript is supported by the supplemental data presented.

We thank you for your interest in and support of The Plant Cell. We hope we will be able to render a more positive decision on future work.

Sincerely,

The Plant Cell Editorial Board

Dear Editors,

In this revision, we have made a thorough effort to address all the concerns and suggestions raised by the reviewers. Specifically, we have prepared a whole-genome crossover map of Col×Ler hybrid based on 238 F_2 individuals, significantly

increased the number of recombinants in the seed-typing analysis for the *ChP* interval (from 68 to 142), and included negative control for the gammaH2A.X analysis (*spo11-1* mutant).

After carefully reviewing the feedback, we concluded that the analysis of pseudo-hotspots presented in the original version of the manuscript relied on overly broad generalizations and raised too many concerns within the scientific community to retain it in the paper. Therefore, we have decided to remove this section from the manuscript.

However, we obtained new and intriguing results regarding genetic interactions in combinations of the *atr* mutant with *zyp1* and *fancd2* mutants, which we have now included in the study. We discovered that while the effect observed in *atr* is independent of the activities of both ZYP1 and FANCD2 proteins, the double mutants *atr zyp1* and *atr fancd2* behave differently: In *atr zyp1*, both mutations act synergistically, enhancing the crossover redistribution effect. In contrast, no further enhancement is observed in *atr fancd2*, suggesting that the inactivation of these two genes triggers a common mechanism of crossover redistribution (though initiated through independent pathways). Although the mechanism remains unclear for both genes (Kurzbauer et al., Plant Cell 2012; Kurzbauer et al. Plant Cell 2018, Li et al., PNAS 2021), we believe that our new results shed light on the functions of ATR significantly contributing to our understanding of the role of this kinase in meiosis.

-- Reviewer comments:

Reviewer #1 (Comments for the Author):

Longfei Zhu and authors present their results on the characterization of the meiosis behavior of the atr mutant in Arabidopsis. In a previous paper (Zhu et al., 2021), they had identified an increase in the recombination frequency at the '420' locus in the sni1 atr double mutant compared to the single sni1 mutant, and in this paper, they characterize further the effect of atr on the recombination landscape. The paper is well written and the figures are nicely laid out.

Thank you for appreciating our characterization of the *atr* mutant and the way the manuscript was prepared.

While no difference in the number of DSBs is observed (based on gH2AX staining), more crossovers are observed using fluorescent markers segregation-based measures in intra-arm intervals, while less crossovers are observed in intervals closers to the centromeres. This delocalization of crossover events seems to affect both class I and class II crossovers, as the two pathways are both required for and affect by this effect. This suggests that ATR regulates recombination steps upstream of class I/class II actors, probably at the invasion step.

My major comment is in regard to the use of appropriate controls for both the whole genome and the hotpost crossover detection by sequencing experiments. In both cases, the authors generated the atr data de novo for the paper, but did not generate any new wild-type data (sisters of the atr mutant), and instead used previously published data (Rowan et al, 2019 for the genome wide data and their own published data for the hotspots Szymanska-Lejman et al., 2023). This is concerning, and I invite the authors to justify this choice thoroughly. Although I understand that whole-genome sequencing experiments are expensive, I would strongly advise the authors to consider sequencing the F2 descendants of the wild-type sister plants of their atr mutant grown in the same controlled environment, and include these new data in the paper as a proper control. I have to request at the very least sequencing of wild-type sister recombinant ESILs lines for the data included in Figure 3E (blue wild-type data). All meiotic mutant lines are best kept as heterozygous seed stocks, so the authors should have access to the sister wild-type plants. I understand that it requires crosses that might not have been performed at the time, but I really strongly encourage the authors to go back and generate the appropriate material for proper comparison.

We agree with the reviewer that appropriate controls for experiments are crucial. However, it should be noted that previous comparisons of chromosomal crossover distribution in the wild-type Col×L*er* did not show any statistically significant differences (see, for instance, Blackwell et al. 2020). Nevertheless, we decided it was worthwhile to generate a new crossover map based on GBS for the Col×L*er* cross because in the initial version of our manuscript we used data developed by another team, and not all plant growth parameters can be replicated (Rowan et al. 2019). Our crossover map includes a total of 238 individuals and 1922 crossovers, and it does not differ significantly from the Rowan et al. 2019 map (comparison of maps below). Therefore, all conclusions drawn from the comparison between the *atr* mutant and the wild type have been preserved.

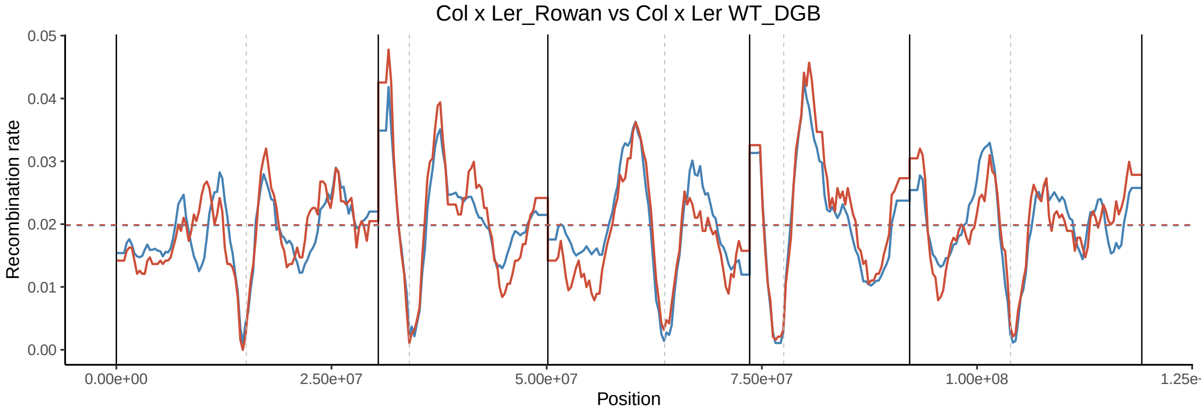

However, we decided not to repeat the control for the seed-typing analysis for the ChP interval. The main reason for not conducting this analysis is the fact that the wild-type plants used in the ChP experiment published in Szymanska-Lejman et al. 2023 were grown at the same time and under identical conditions (the same growth

chamber) as the plants used in the *atr* experiment. Instead, we decided to strengthen this part of the results by expanding the seed-typing analysis for *atr*, increasing its scale from 68 individuals to a total of 142 individuals. As a result, the quality of our information regarding recombination in the *atr* mutant in the ChP interval is now significantly higher, and the results of the analysis are more convincing.

Points of further discussion:

- How would you explain that we do not see a difference in recombination frequency at the ChP and BT hotspots in a pure Col background (Fig 3B)? You discuss the difference between Col/Col and Col/Ler in the context of the MLH1 focus count in your discussion, but I don't recall any discussion of this figure.

Indeed, this is a result that we did not discuss in the paper, because we believe that this observation is not directly related to the effects of ATR, but rather is a result of the specific properties of the experimental set-up we used. The ESIL intervals are very short; ChP is 26.3 kb and BT is 49.1 kb, encompassing only a few recombination hotspots, and therefore are well-suited for studying hotspot activity. At the same time, it is unlikely that recombination in such a small segment of the chromosome, representing only 0.11% and 0.21% of the chromosome respectively, will illustrate crossover changes on a chromosome scale. We assume that there are significant differences between individual hotspots and their activity may be regulated in different ways. For example, crossover interference, which is active in the *atr* mutant, may contribute to the local lack of change in the mutant compared to the wild type. Therefore, we use recombination measurement in such short intervals only to examine to what extent the hotspots they represent are susceptible to a given mutation. Based on the results presented in Fig. 3B and 3C, we decided that the analysis of hotspot activity is justified for ChP, as this interval shows a difference in activity in the hybrid atr compared to wild-type hybrid.

- Similarly, why do we see a difference between WT and atr in Col/Ler context at the ChP hotspot but not at the BT one?

Also in this case, we think that this is an effect resulting from the length of the intervals. However, changes in ChP recombination frequency in Col/L*er* hybrids and their absence in Col/Col inbreds may also be due to a combination of recombination modifiers differing between Col and L*er* (*HEI10* and *SNI1*). Specifically, *SNI1*^{Ler} behaves similarly to the hypomorphic *sni1-1* allele, which causes an increase in crossover frequency in the chromosome arms but a decrease in pericentromeric regions (Zhu et al. 2021). The combined effect of *SNI1*^{Ler} and *atr* inactivation may lead to a noticeable decrease in crossover frequency in the pericentromeric ChP in hybrids compared to inbreds. Since we consider these considerations to be highly speculative, we did not include them in the discussion.

Minor comments:

Figure 1

- Any statistical correction for multiple testing? I doubt that it will affect the conclusions at all, but it is good practice. You can additionally remove the exact p-value numbers (which are not very meaningful anyway) and replace them with **/*** when reaching appropriate thresholds

As suggested, we applied a correction for multiple testing for data shown in Figures 1B-D. To determine statistical significance, we used one-way ANOVA followed by Tukey HSD. For Figures 1E-F, we retained the Welch t-test, as there are only single comparisons.

- I would appreciate a simple scheme to indicate the location on chromosomes 1 and 3 of all measured intervals (with appropriate scale, see below Figure 3A).

Thank you for this suggestion – we have included ideograms of chromosomes 1 and 3 illustrating the location of the intervals used to measure crossover rate in Figure 1B.

- '420' is jargon for anyone but us! Please use appropriate wording in figure legends.

Corrected.

Figure 2

- Legend: what is the window size for binning crossover events? (SNPs is 300kb)

This is the same as for SNPs, i.e., 300 kb. We added appropriate information to the figure legend.

Figure 3

- Fig 3A: Please add a scale (mm to Mb) or exact positions (in Mb, if the scheme is up to scale) of CEN, BT, ChP and pericentromeric boxes.

Done!

- What definition of "pericentromeric" did the authors choose? Based on what kind of data?

We adopted the definition from Choi et al., 2018, according to which the pericentromeres are defined as regions with higher than average DNA methylation, which surround the centromeres (Choi et al., 2018). We include this information in Fig. 1 & 3 legends.

- Fig 3E: could you move the names of the hotspots (cute dogs as they are) below the position in Mb of the x axis? It looks very crowded over imposed on the graph itself.

Done

- Fig 3F: I would advise using stacked bar graph, as you are comparing proportions of events in all three categories.

Changed as requested!

- Fig 3D is barely mentioned in the text, it could be moved to supplementary

Although this illustration is not widely discussed in the text, we believe it should remain in the main figure rather than in the supplementary data, as it provides information about the resolution of CO mapping in individual recombinants. Additionally, TPC limits the use of supplementary figures mainly to large-scale datasets and detailed experimental protocols.

- Fig 3G: same comment for multiple testing and p-values as in Fig 1.

We applied Kruskal-Wallis test followed by post-hoc Dunn's test, as the sample did not followed normal distribution. This test corrects for multiple comparisons (Bonferroni correction).

Figure 4

- Fig 4A: I am not sure I understand this figure. Are you comparing the 52k crossover events in the wild type to your much smaller data set in atr? Are you normalizing for the total number of crossovers? I would guess not because the atr mutant shows a bit more crossovers than the wild type based on Fig 2B. I think this "pseudohotspot" is an interesting approach, but I am not sure this statistics and this representation is the most suitable to convey your point.

In the new version of the manuscript, due to criticism of the approach by other reviewers, we abandoned this analysis and therefore the entire figure was removed.

Figure 5

- Legend reads "crossover interference is weaker" although all comparisons are non-significant and the text actually says page 13 line 337 that "interference is not affected in atr"

Thank you for noticing this mistake, and we apologize for it. In the very first version of the manuscript, we had fewer reads for *atr*, and initially, it appeared that the mutant exhibited reduced interference. However, our data ultimately did not confirm this conclusion. We mistakenly retained the old version of the figure title.

- There is no figure 5F (anymore?), although the legend still mentions it. I think you can just remove the legend part, the interference data from FTL on chromosome 3 are not adding anything more I guess (no difference, or maybe a slight one?)

In the original version of the manuscript, we presented results for FTL *I3bc* suggesting a slight decrease in interference. However, we decided to remove them because they were of low quality. Unfortunately, we currently do not have access to a flow cytometer that allows for precise measurement of interference. Given the lack of a decrease in cis-inter crossover distances, we concluded that it does not make sense to use FTL for a single interval in this project.

Figure 6

- Maybe you could consider merging Figure 5 and 6 to convey the point that both pathways are similarly affected by the atr effect? You could move panels 5E and 6C to supplementary. This is a proposition and a personal preference; the authors are free to not consider it.

We have added a negative control (*spo11-1*) to Figure 5A. It seems to us that moving Fig. 5E and 6C to the supplement is not possible due to the mentioned TPC restrictions ror supplementary information. Moreover, the results presented in 5E are important, indicating the lack of any serious interference disorder in *atr*. Therefore, we decided to leave the previous arrangement of figures.

Figure 7

- Legend: title should read "remodeling of crossover distribution" (the distribution is remodeled, not the crossovers)

Corrected!

- P-values: see above

Wherever possible, we used correction for multiple comparisons.

Figure 8

- P-values: see above

Wherever possible, we used correction for multiple comparisons.

Figure 9

- For which comparisons are the p-values? To wild type? To avoid over-crowding of the diagram, you could mention in the legend that "all comparisons are statistically significant (compared to xx) except when noted"

Thank you for this comment. We have clarified what the *p*-values refer to.

Main text

- Page 3 line 69-74: I am of the opinion that not all second-end captures give rise to nice and simple dHj (see de Muyt et al., 2012 for instance). Moreover, we have no way of knowing whether "a majority" of D-loops are dissolved through SDSA. What we do know is that there are more complex events that we first considered (see Ahuja et al., 2021), so a lot might be happening at the invasion step. Most D-loop are converted to non-crossovers (or inter-sister events), that much we know. Please reword this bit.

We thank the reviewer for these valuable comments. We have revised the text accordingly to incorporate them.

- In the last paragraph of the introduction, you already mention pseudo-hotspots. I was not familiar with this approach before reading your manuscript, and it felt weird reading about it at this time in the text. I would say less at this point (trying to word it without needing to use the pseudo-hotspots?) and keep the full description for the results section.

This fragment was removed from the introduction.

- If you choose to merge figure 5 and 6, you could merge the respective paragraphs as well.

Ultimately, we did not merge these figures; therefore, for clarity, we kept the paragraphs separate.

- I do not like the GBS acronym. It is not a well-known acronym, and I would refrain from using it extensively (use "sequencing data" maybe?). This is a personal preference.

We replaced it with the term “genome sequencing”.

- Please italicize Arabidopsis everywhere, it is a species name.

In our work, we use the word "Arabidopsis" as a common name, not as the species name, which is *Arabidopsis thaliana*. Writing "*Arabidopsis*" in italics refers to the genus name *Arabidopsis*, which would be justified if we were studying different species, which we are not. Therefore, we would like to stick with the non-italicized spelling "Arabidopsis," as it is widely accepted in the Plant Cell.

- Careful about atr not being properly italicized either sometimes (e.g. line 275).

Corrected.

- Line 385: the gene msh4 should be in small caps

Corrected.

I liked this paper and I hope the authors will have the resources to provide us with appropriate controls that will allow their conclusions to be supported accordingly.

Best of luck for the revisions.

We are pleased that the reviewer liked the manuscript and we thank you very much for this very insightful and constructive review.

Reviewer #2 (Comments for the Author):

In this manuscript, Zhu et al explore the role of the ATR kinase in meiotic crossover formation in Arabidopsis. They conclude that ATR inactivation leads to the redistribution of crossover along chromosomes, with a decrease in centromere-proximal regions and an increase in distal regions. Some interesting results are presented, however, the main conclusions are insufficiently supported as detailed below:

Major comments:

1) In all genome-wide analyses, the atr Col/Ler hybrid is compared with a col/ler wild type previously described and produced in a different lab, without internal wild type controls. This is problematic as environmental factors or potential differences in the genetic background could affect crossover number and distribution. The author must include an analysis of the Col/Ler wild type from the same lineage and in their lab conditions.

We thank the reviewer for this important point, with which we agree. For the purposes of this work, we have prepared a new crossover map based on sequencing of 238 F_2 individuals from a Col×Ler cross grown under identical conditions as those for the *atr* mutant. Our control does not differ significantly from the data previously used in the work of Rowan et al. 2019 (see a figure added in the response to the first reviewer). Therefore, the conclusions from the comparison of the *atr* mutant with the wild type did not change.

2) The authors use immunodetection of γH2A.X to quantify DSB formation. However, the specificity of the staining/antibody is not supported. A negative control is essential. One possibility is to use a known DSB defective mutant (e.g. spo11) and show that, when done in parallel, foci are observed in wild types and not in mutants.

Thank you for this comment. To confirm the specificity of the antibodies used in the experiment, we conducted the control suggested by the reviewer under identical growth conditions using the *spo11-1* mutant (new panel in Figure 5A). This experiment confirmed that the antibodies used correctly recognize DSB sites.

3) Line 238-250. While it is clear that all three hotspots have reduced activity I'm not convinced that there is any significant difference in the contribution of the different hotspots. A proper test should be performed (e.g. Chi-square) to test if the proportion of CO in the three hotspots is different between wild-type and mutant. With n=68, my prediction is that this will not be significantly different. All parts of the manuscript using this conclusion (differences in hotspot usage) should be revised.

We agree with the reviewer that 68 recombinants for *atr* may be too small a sample size to estimate differences between the wild type and the mutant. Therefore, we went back to our materials and selected an additional 74 recombinants, which we examined using seed-typing. In total, we identified COs for 142 *atr* plants, providing

a sufficient number to reliably compare hotspot activity. A comparison of crossover distribution in three hotspots in atr relative to wild type using the Chi-square test did not show significant differences. However, we additionally analyzed the relative changes in activity of individual hotspots in *atr* compared to wild type by examining individual SNP-SNP sections for each hotspot (Fig. 3G). For this analysis, we used the nonparametric Kruskal-Wallis test followed by post-hoc Dunn's test with Bonferroni correction, which revealed a significant difference between the strongest hotspot (*Coco*) and the weakest hotspot (*Aro*). Based on these observations, we concluded that while overall hotspot usage is not significantly different between atr and wild type, the activity of individual hotspots may vary. In the revised manuscript, we have tempered the conclusions of this analysis accordingly.

4) L251-277. I'm not convinced of the pertinence of this analysis. First, this is not an analysis of hot spots (as recognized by the usage of the word „pseudo-hotspot" in the text). So the conclusion of „biased hotspot usage" is not supported. At best the analysis indicates how clustered are the COs. Second, the observed effects are quite subtle. For example, 11.7% vs 12.8% of crossover found in the „extremely strong category) is not a large effect. Figure 3A should show the % rather than numbers (and Figure 4B could be omitted). Third, and probably more importantly, I'm afraid that the method could be biased by the fact that the will-type data is a very large part of the „reference data" that defines the „hotspots". Thus relatively cold intervals could have by chance one rare crossover in wild-type, and the interval would be classified „sporadic". Then, the same CO would be counted as falling in a „sporadic" interval. This effect could inflate the proportion of „sporadic" CO in the wild type, and then largely explain the observations. Finally, and as mentioned in the first point the mutant and wild-type data were produced in different conditions.

After considering the opinions of all three reviewers, we concluded that our proposed aggregate pseudo-hotspot analysis may be biased and therefore decided to abandon it.

5) L289. How many cells were analyzed?

We analyzed 134 cells in the first meiotic division and 177 in the second meiotic division (a total of 311 meiocytes) from three different plants. This information has been included in the revised manuscript.

Minor comments

It would be useful to comment on fertility in atr. Reduction of fertility does not necessarily mean a meiotic defect, but full fertility (as shown later in the manuscript) means that meiosis is fine.

We added this point to the first paragraph of the discssion.

In the introduction, the authors navigate between the conserved features of meiosis recombination and focus on arabidopsis. This is fine, but it should be always clear what we are talking about. For example, lines 94-97 this statement and numbers are for Arabidopsis, but this is unclear and should be clearly stated. Please review the entire introduction with this in mind.

In the quoted sentence, we indicate that it refers to *A. thaliana*. However, we reviewed the entire manuscript, adding species information where necessary.

Line 180. There is a discrepancy in the number of analysed plants between the figure (n=180) and the text (n=220)

We apologize for this oversight, which resulted from the use of a scheme initially prepared for a different genotype, where we used a smaller number of samples. For *atr*, we used 220 individuals as described in the text.

223-224. Please clarify: how amplified? how sequenced?

We included more information in the manuscript text. Briefly, for each recombinant we amplified the entire region located between the reporters using high-fidelity long-range PCR (with three overlapping amplicons of 8-10 kb), pooled the PCR products and prepared libraries with separate barcodes. In this way, each recombinant was represented by a separate library. The libraries were then pooled and sequenced using HiSeqX-10 to a depth of approximately 1500× per library. After demultiplexing, we identified the crossover sites based on SNPs distinguishing Col from L*er*. We determined the topography of crossovers along the interval by summing the data for all recombinants. Below is an example graph showing the identification of the crossover site in a single *atr* Col/L*er* recombinant, where each dot represents a SNP site, Col SNPs are shown in blue and L*er* SNPs in red:

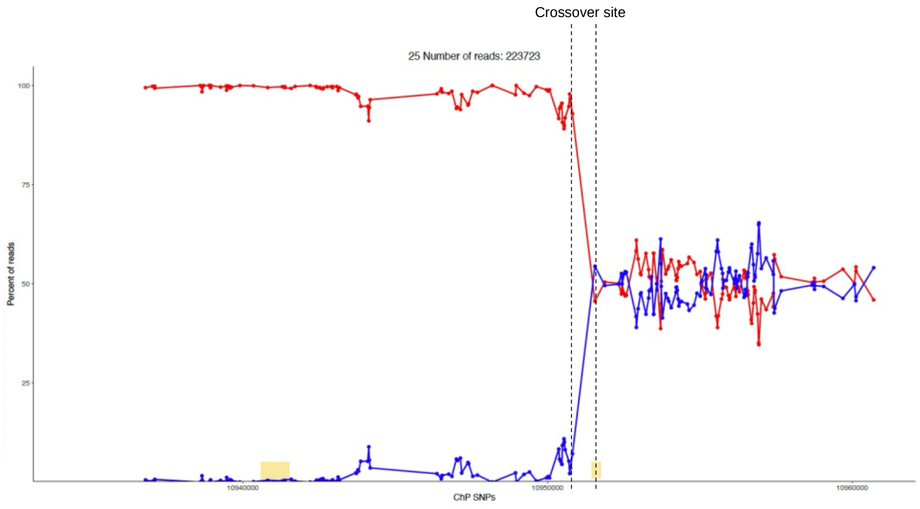

Figure 2E. What is shown in blue/green/red on the top of the graph?

These correspond to positions of genes (light-green (forward) and dark-green (reverse) rectangles) and transposons (blue rectangles). The description has been added to the figure legend.

Please provide the catalog number of the γH2A.X antibody

We used Ab from Upstate, #05-636 (link below). The proper information has been added to the Methods section.
https://www.sigmaaldrich.com/ES/es/product/mm/05636?icid=sharepdp-clipboard-copy-productdetailpage

L328. The distribution of class II CO is not random. Independent would be more appropriate.

We apologize for this imprecise expression. We corrected it as suggested.

L343. Is there any reliable Class II crossover markers in non-plant eukaryotes?

Good point! We removed “for plants” from the sentence.

L352. Neither an increase nor reduction in class II COs.

Corrected as suggested.

Reviewer #3 (Comments for the Author):

This study performs the genetic analysis to better understand the function of Ataxia telangiectasia-mutated and Rad3-related (ATR) kinase in meiotic recombination in Arabidopsis thaliana. ATR kinase in Arabidopsis had been shown to regulate the deposition of DMC1 at meiotic resected breaks but did not appear to affect meiosis overall in a drastic manner unless combined with ATM kinase. The manuscript submitted by Zhu et al. presents a detailed description of the role of ATR kinase in controlling the distribution of meiotic crossovers. The conclusions of the paper are as follows. In the atr mutant, the CO frequency is increased and there is a redistribution with a CO increase in the subtelomeric region and a decrease in the pericentromeric region, the hotspot usage is modified. However, the authors do not find any correlation or causative link between this change in CO distribution and heterozygosity along the chromosome, number of meiotic DSBs, interference measured with MLH1 foci representing class I CO or with components of class I or class II pathways. Overall, this study concludes that the loss of ATR is manifested in the redistribution of CO and hotspot usage, which are attributed to the likely early role of ATR in meiotic recombination. There is a good quantity of data in this paper

and the experiments are carried out using well-established methods combining genetics, genomics, and cytology approaches with proper design.

We would like to thank the reviewer for appreciating the amount of work we put into preparing this manuscript.

Major concerns:

However, the study remains descriptive without any molecular mechanism identifying how ATR might be controlling the distribution of CO.

It is true that, despite our broad and versatile characterization, the exact mechanism of ATR action on meiotic recombination has not been elucidated. However, we note that in this work we find for the first time that *atr* mutants show a strongly altered crossover distribution. In addition, we verify a number of hypotheses regarding what this effect could be caused by - ranging from controlling the number of DSBs, to extensive analyzes of genetic interactions (measuring crossover frequency in combinations with *sni1*, *zyp1*, *zip4*, *mus81*, *fancm*, *fancd2*). In the revised version of the manuscript, we present new data on the combination of the *atr* mutant with *fancd2* and *zyp1*. We point out that while the *atr* effect is independent of both of these factors, the crossover redistribution mechanism in *atr* may be similar to that in *fancd2*. At the same time, *atr* and *zyp1* act synergistically, which indicates separate mechanisms of functioning. We believe that these new results shed new light on the functions of ATR in meiosis.

1. The claim that ATR controls the number of COs is not evident. Indeed, genome-wide increase of CO is not very evident except in chromosome 3 and to a small extent in chromosome 5, other chromosomes don't show an increase in CO frequency. It is also not very clear if this redistribution of CO is very specific to ATR or if it is a general phenomenon of mutants affecting CO formation. A similar redistribution is observed in asy1+/- heterozygous (Lambing et al 2020 PNAS) and figl1 mutants (Fernandes et al 2018 PNAS).

In the paper, we do not claim that the number of crossovers is increased in *atr*. Our main conclusion concerns the redistribution of crossovers along the chromosome. While we agree with the reviewer that the redistribution profile we observed appears in some mutants, it is difficult to assume that a mutation in any gene in *Arabidopsis* always leads to the same changes in crossover distribution. Moreover, the scale and extent of these changes vary among different mutants, and the underlying mechanisms are likely distinct as well. In *asy1/+*, the mechanism is likely based on the structural role of ASY1 in meiotic chromosome formation, but in *figl1*, the mechanism remains far from understood, despite numerous reports on the subject.

In the newly added experiments in our study, we show that while the effect of *atr* on crossover redistribution is independent of the presence of FANCD2, the mechanism

itself may be similar, as the double mutants exhibit characteristics of the single mutants. This is different in the case of *zyp1*, where the double mutant shows an enhancement of the effect.

2. Furthermore, the interference analysis based on MLH1 foci count done at the pachytene stage may not be the best stage when looking for redistribution and may not provide a true representation of chiasma. The dynamics of MLH1 foci has been shown to be modified between Pachytene and Diplotene or Diakinesis and It would be better to look at these later stages (Lambing et al 2020 PNAS, and Li et al 2021 PNAS (HEI10 foci)), where one can measure true redistribution per bivalent and a putative mutant effect on the dynamics of the MLH1 foci.

In fact, we did not analyze interference based on MLH1 foci. The results shown in Figure 4E refer to cis-intercrossover distances measured through GBS analysis (combinations of genotypes CC-CL-CC and LL-CL-LL). Since we observed no significant changes in the length of these distances in *atr* compared to wild type, apart from a small difference on chromosome 1, it seems very unlikely that interference is significantly reduced in *atr*. For these reasons, we decided against conducting an analysis based on the distribution of MLH1/HEI10 foci, which requires advanced microscopy equipment and is technically very challenging. It's worth noting that in the current version of the paper, we replaced the wild type data from Rowan et al. (2019) with results obtained in our laboratory from Col×L*er* plants grown under identical conditions as the *atr* mutant. This makes our findings even more compelling and indicates no significant changes in crossover interference in *atr*.

3. The proposed model for ATR controlling the hotspot usage remains rather correlative than causative. The study should have least included an analysis of the localization of the hotspot regarding their strength. Also, it would have been interesting to analyze the hotspot usage in asy1+/- and figl1 mutant with a similar approach.

Since other reviewers also criticized this research approach, suggesting that it may lead to false conclusions, we decided to abandon this analysis.

The results provided here are likely to be interesting for the meiotic recombination community, but conclusions made possible by the data are of insufficient impact to justify publication in the Plant Cell as compared to a more specialized journal.

With respect to the reviewer, we disagree with this opinion. We are the first to show that a mutation in ATR, one of the two key DNA damage-sensing kinases, leads to significant crossover redistribution across the entire genome. Such an observation has not been made before, not only in plants but in any eukaryotes. We then present a comprehensive set of experiments using the most advanced techniques available to characterize the impact of the *atr* mutation at various stages of recombination, starting from DSB formation, through genome-wide and hotspot-specific crossover mapping. We also examine the progression of meiosis and any associated

disturbances, analyze the *atr* mutation in the context of both crossover pathways in Arabidopsis, in relation to DNA polymorphisms between homologs, and with reference to numerous meiotic recombination regulators acting at different levels and stages, such as FANCM, MUS81, FANCD2, and ZYP1. In doing so, we have ruled out several hypotheses about the role of ATR in recombination and significantly expanded the understanding of its function, providing further insights into its mechanism of action.

1st Revision - Editorial Decision 09-Sep-2024

Dear Piotr,

We have received reviews of your manuscript entitled "ATR controls meiotic crossover distribution at the genome scale in Arabidopsis." On the basis of the advice received, the board of reviewing editors would like to accept your manuscript for publication in The Plant Cell. This acceptance is contingent on revision based on the comments of our reviewers. In particular, please consider to provide the statistics supporting the shift in CO distribution in the whole genome CO maps.

Please highlight all changes and include a detailed annotation of changes of the text, with line numbers, and noting your responses to the comments.

To submit your revised manuscript, click:

Link Not Available

If you have any questions about the revision submission procedures, please contact the Editorial Office Staff (tpc-submissions@aspb.org). If you cannot return the revised manuscript within 30 days, please let us know. Otherwise, we will assume that you have elected not to revise the manuscript and withdraw it.

Thank you very much for the privilege of reviewing this work. I look forward to receiving the next version.

Sincerely,

The Plant Cell Editorial Board

Reviewer #1 (Comments for the Author):

The authors have answered most if not all of my comments.
I have one more major comment:
- Page 9 line 210: "clear redistribution of COs from pericentromeric regions to interstitial and subtelomeric regions".
Could you provide some statistics to support this claim? Either on the aggregated data (Fig 2C) or chromosome by chromosome (Fig 2D).

Thank you for all your hard work.
All the best

Reviewer #2 (Comments for the Author):

The authors have satisfactorily addressed my concerns.

minor comments:

- The author should specify that "cis-DCO" are P1-HET-P1 (P1-hetP2 are trans-CO). By the way, it could be interesting to analyze in parallel the "trans-CO", which would be a good control for the absence of interference.
- L495. "appears to be universal" would be more appropriate.

Please also note the following:

-The Plant Cell now requires authors to complete and submit an author revisions checklist upon submission of a revised

manuscript. The aim of the checklist is to aid authors in preparing a high-quality manuscript, facilitate the review and assessment of revised manuscripts, and help to ensure that journal standards are maintained across the board. If your manuscript is accepted, the completed checklist will be published as supplemental material attached to the article online. Please download a copy of the checklist (pdf fillable form) at this link, for submission with your revised manuscript: https://tpc.msubmit.net/html/Author_Revisions_Checklist.pdf.

-Supplemental materials should be restricted to large datasets and tables, presentation of replicates, and validation of reagents, methods, or genotypes. Any data that are used to support the major claims must be in the main manuscript. Supplemental figure legends must indicate what figure in the main manuscript is supported by the supplemental data presented. Please justify how each of the supplemental figures meet the criteria.

-Sampling methods and the nature of "biological replicates" should be described precisely (i.e. different plants, parts of plants, pooled tissue, independent pools of tissue, sampled at different times, etc.), along with a clear description of and rationale for any statistical analyses conducted. The reader should know exactly what was sampled; what forms the basis of the calculation of any means and statistical parameters reported. This is also necessary to ensure that proper statistical analysis was conducted.

Responses to Reviewers' comments:

Reviewer #1 (Comments for the Author):

The authors have answered most if not all of my comments.

I have one more major comment:

- Page 9 line 210: "clear redistribution of COs from pericentromeric regions to interstitial and subtelomeric regions".

Could you provide some statistics to support this claim? Either on the aggregated data (Fig 2C) or chromosome by chromosome (Fig 2D).

We agree with the reviewer that demonstrating statistical differences in crossover frequency between the two genotypes across regions is important. We performed a cumulative analysis, which shows a statistically significant difference between *atr* and wild type for both chromosome arm and pericentromeres. These results are presented in the newly added Figure 2E. The corresponding text has been added in lines 210-214.

Thank you for all your hard work.

All the best

Reviewer #2 (Comments for the Author):

The authors have satisfactorily addressed my concerns.

minor comments:

- The author should specify that "cis-DCO" are P1-HET-P1 (P1-hetP2 are trans-CO). By the way, it could be interesting to analyze in parallel the "trans-CO", which would be a good control for the absence of interference.

Thank you for this important point! As requested by the reviewer, we have clarified that cis-DCO refers to Col-HET-Col or Ler-HET-Ler combinations (line 322). However, we did not conduct a trans-DCO analysis because we believe such an analysis would only be meaningful if we had demonstrated differences in cis-DCO distance lengths between the *atr* mutant and wild type, which we did not observe.

- L495. "appears to be universal" would be more appropriate.

Corrected as requested (line 499).

Dear Piotr:

We are pleased to inform you that your paper entitled "ATR controls meiotic crossover distribution at the genome scale in Arabidopsis" has been accepted for publication in The Plant Cell, pending a final editorial review by a science editor. At this stage, your manuscript will be evaluated by a science editor with respect to its presentation of scientific content, compliance with journal policies, and presentation for a broad readership. The Plant Cell has contracted with Plant Editors (planteditors.com) to provide this service to our authors, and you will soon receive additional information on this process.

Please note that each author needs to link their ORCID identifier to their account in the system before your manuscript can be published. If any authors do not have an ORCID linked to their account, they will receive a message with a link to complete this task. Please ensure that ALL of your coauthors have completed this task as soon as possible.

LICENSE INFORMATION Shortly after your paper is sent to production, you will receive an email from our publisher's author support team, **SciPris**, with information on article processing charges, as well as the types of Open Access license you would be able to purchase. The accepted version of your manuscript will not appear on our Advance Articles page until the license is selected and signed, so please look out for an email from SciPris. For more information see the Article Publication Fees section of our author guidelines: https://academic.oup.com/plcell/pages/General-Instructions

The Plant Cell and The Arabidopsis Information Resource (TAIR) are collaborating to collect functional annotation data about Arabidopsis genes from authors. This includes information about the gene's molecular function (e.g., kinase activity, ATP synthetase activity), the biological process/es it is involved in (e.g., endosperm development, threonine biosynthesis), its subcellular location (e.g., nucleus, ER), anatomical or developmental expression pattern (e.g., leaf, ovule, flower stage 10, seedling stage), or its partner in a protein-protein interaction (e.g., AT1G01010 interacts with AT1G01020).

If your paper contains results falling into one or more of these categories for Arabidopsis genes, we request that you now submit these data for inclusion in TAIR by filling in the form provided at the following URL: https://www.arabidopsis.org/doc/submit/functional_annotation/123. If you need further clarification on what types of data can be submitted please contact curator@arabidopsis.org.

Finally, we encourage your submission of artwork for the journal cover. If there is an image or illustration related to your paper that you would like to have considered, please email it in .pdf or .tif format to clowe@aspb.org. An image title, brief, 2-3 sentence description of the image, and the name of the person credited for the image are also encouraged. If your image is selected to appear on the online cover of the journal, you will be contacted with the scheduled issue date and further information.

We look forward to seeing your paper published.

Sincerely,

The Plant Cell Editorial Board

=====
IMPORTANT REMINDER: PEER REVIEW REPORTS
=====

If you opted to publish a peer review report along with your article during the original submission process, it will be prepared by the editorial staff and publicly posted with your manuscript, inside the zip file that contains any other supplemental material. As a reminder, the peer review report is a public record of all comments from editors and reviewers, as well as your prior responses, as you received them in the decision letters for each draft of your manuscript. If you agreed to publish this report and have changed your mind, or are not sure if you selected this option, please contact the editorial office as soon as possible before signing the license agreement from our publisher.

=====

----FOR ASPB OFFICE USE ONLY (DO NOT EDIT)----
MSID: 40545

Corresponding Author: Prof. Ziolkowski
